# Heterologous Expression and Assembly of Human TLR Signaling Components in *Saccharomyces cerevisiae*

**DOI:** 10.3390/biom11111737

**Published:** 2021-11-22

**Authors:** Julia María Coronas-Serna, Elba del Val, Jonathan C. Kagan, María Molina, Víctor J. Cid

**Affiliations:** 1 Departament of Microbiology and Parasitology, Faculty of Pharmacy, Universidad Complutense de Madrid and Instituto Ramón y Cajal de Investigación Sanitaria (IRyCIS), Pza. Ramón y Cajal s/n, 28040 Madrid, Spain; juliacor@ucm.es (J.M.C.-S.); elbadval@ucm.es (E.d.V.); 2 Division of Gastroenterology, Boston Children’s Hospital and Harvard Medical School, Boston, MA 02115, USA; jonathan.kagan@childrens.harvard.edu

**Keywords:** *Saccharomyces cerevisiae*, humanized yeast, innate immunity, ERMES, MyD88, TIRAP, TRAM, TRIF, TIR domain

## Abstract

Toll-like receptor (TLR) signaling is key to detect pathogens and initiating inflammation. Ligand recognition triggers the assembly of supramolecular organizing centers (SMOCs) consisting of large complexes composed of multiple subunits. Building such signaling hubs relies on Toll Interleukin-1 Receptor (TIR) and Death Domain (DD) protein-protein interaction domains. We have expressed TIR domain-containing components of the human myddosome (TIRAP and MyD88) and triffosome (TRAM and TRIF) SMOCs in *Saccharomyces cerevisiae,* as a platform for their study. Interactions between the TLR4 TIR domain, TIRAP, and MyD88 were recapitulated in yeast. Human TIRAP decorated the yeast plasma membrane (PM), except for the bud neck, whereas MyD88 was found at cytoplasmic spots, which were consistent with endoplasmic reticulum (ER)-mitochondria junctions, as evidenced by co-localization with Mmm1 and Mdm34, components of the ER and Mitochondria Encounter Structures (ERMES). The formation of MyD88-TIRAP foci at the yeast PM was reinforced by co-expression of a membrane-bound TLR4 TIR domain. Mutations in essential residues of their TIR domains aborted MyD88 recruitment by TIRAP, but their respective subcellular localizations were unaltered. TRAM and TRIF, however, did not co-localize in yeast. TRAM assembled long PM-bound filaments that were disrupted by co-expression of the TLR4 TIR domain. Our results evidence that the yeast model can be exploited to study the interactions and subcellular localization of human SMOC components in vivo.

## 1. Introduction

Eukaryotic complex organisms have evolved diverse mechanisms to react to the presence of pathogens and discern them from their microbiota. The lipopolysaccharide (LPS) of Gram-negative bacteria is a major pathogen-associated molecular pattern (PAMP). It is recognized by the Toll-Like Receptor 4 (TLR4), leading to the activation of innate immunity signaling. Unlike classic signaling pathways, those downstream PAMP-recognizing receptors (PRR) rarely rely on secondary messengers. Rather, their signaling mechanism depends on the assembly of supramolecular organizing centers (SMOCs) [1]. Upon recognition of low concentrations of their ligands, PRRs oligomerize and amplify the signal by recruiting adaptor proteins to form the SMOC physical scaffold, which will eventually summon effector enzymes, such as kinases, E3 ubiquitin ligases, and proteases [1,2]. The signal, thus conveyed through protein-protein interactions and posttranslational modifications, ends up in nuclear translocation of transcription factors to induce the expression of cytokines, which eventually set off typical innate immunity responses [3,4].

All ten human TLRs require the key component Myeloid Differentiation primary response 88 (MyD88) to convey signals, excepting TLR3, which exclusively uses the Toll Interleukin-1 Receptor (TIR)-containing adaptor inducing interferon-β (IFN-β) (TRIF, a.k.a TICAM-1). TLR4 itself steers alternative signaling pathways through either MyD88 at the plasma membrane (PM) or TRIF at the endosome. The TIR-containing adaptor protein (TIRAP, a.k.a MAL) is an additional adaptor required for TLR4 at the MyD88-dependent pathway, whereas the TIR-domain-containing adaptor molecule (TRAM, a.k.a TICAM2/TIRP) is essential for TLR4 signaling through TRIF-mediated endosomal route [5]. Both signaling pathways eventually activate the canonical nuclear factor κB (NF-κB) to trigger the transcription of inflammatory cytokines, but also turn on other unique routes that lead to distinct effector functions [5].

Upon LPS recognition, TLR4 shifts to PM lipid rafts containing the cluster of differentiation 14 (CD14) protein and dimerizes, encountering co-adaptors located in the inner leaflet of those PM microdomains [6]. TIRAP has N-terminal Lys residues that bind phosphatidylinositol(4,5)*bis*-phosphate [PtdIns(4,5)P_2_], an abundant phosphoinositide in PM microdomains involved in signaling [7,8]. TLR4 dimers bind to TIRAP, which in turn recruits MyD88. All these interactions are mediated by a common protein-protein interaction module, the TIR domain. TIR-TIR protein interactions end up in highly ordered hetero-oligomerization forming a left-handed helix [9,10], thus building the SMOC named myddosome, whose stoichiometry is still controversial [9,10]. MyD88 is composed of a C-terminal TIR and an N-terminal Death Domain (DD), which is a different protein-protein interaction module that recruits DD-containing Interleukin-1 Receptor-Associated Kinases1/2/4 (IRAK1/2/4) [11,12]. Thus, myddosome assembly places IRAK kinase subunits close enough to each other to undergo autophosphorylation and activation [13], which in turn leads to recruitment of E3-ubiquitin ligases and downstream effector proteins that eventually transduce the signal to activate NF-κB-dependent transcription [14].

Shortly after triggering MyD88-dependent signaling, the TLR4 complex is internalized by endocytosis [15,16,17]. Low PtdIns(4,5)P_2_ at endosomal membranes disassembles TIRAP and the myddosome [18], switching LPS-bound TLR4 to the TRIF-dependent pathway, where it uses TRAM as its key adaptor [16]. Besides its TIR domain, TRAM has a bipartite sorting region consisting of a myristoylation signal and a polybasic region that targets it to the PM, Golgi, and early endosomes at the resting state [18,19]. TRAM binds the TLR4-TIR domain on the inner side of PM and follows its internalization. Once in early endosomes, TRAM tethers TRIF to initiate the formation of the triffosome SMOC [20]. As in the case of MyD88, homo-oligomerized TRIF is required to activate downstream factors, involving ubiquitin ligases and other downstream components leading to the eventual activation of the interferon regulator factor-3 (IRF-3) transcription factor, which triggers the expression of type I Interferons (IFNs) and TNFα [17,20,21,22].

TLR4 signaling is an important target for the development of immunomodulatory drugs. Agonists of this pathway can be used as vaccine adjuvants or in cancer immunotherapy, while antagonists may be useful as anti-inflammatory drugs against multiple autoimmune pathologies or neurodegenerative diseases [23]. Thus, the development of feasible models for its study is a current demand. Most of the knowledge in the field has been inferred from the properties of heterologous signaling components in animal models, especially mice. Here we evaluate the heterologous expression of human components of TLR4-driven SMOCs in the yeast model organism *S. cerevisiae*. Humanized yeast models, like the one we preliminarily explore here, have contributed along the latest decades to the elucidation of molecular mechanisms, as well as to the development of powerful tools for genetic and pharmacologic screens [24,25,26,27,28,29,30]. Despite having separately evolved over hundreds of millions of years, human and yeast pathways and physiological processes share considerable homology. For example, in a systematic study performed on essential genes, 47% of the human orthologs were able to complement the corresponding yeast deletion mutants [31]. But even when higher eukaryotic pathways are not conserved in *S. cerevisiae*, like those of innate immune signaling, the yeast cell provides a synthetic biology platform for studying their properties in vivo. Thus, heterologous expression of SMOC components in yeast may provide alternative tools for functional approaches. We show that TIR-TIR interactions among human TLR4 SMOC signaling components can be reproduced in *S. cerevisiae*. Furthermore, the localization of myddosome and triffosome components to particular cellular membranes in the yeast model can provide clues for their regulation and function. For example, MyD88 has been described to localize in cytoplasmic spots in mammalian cells, but the nature of these spots has not been elucidated. We show that, in yeast, MyD88 spots correspond to the mitochondria-ER junction complex named ERMES.

## 2. Materials and Methods

### 2.1. Strains, Culture Media, and Growth Conditions

The *S. cerevisiae* strains used in this study are listed in Supplementary Table S1. Standard PCR-based techniques were used to generate the EVY3, EVY4, and EVY5 yeast strains [32]. The monomeric Neon Green fluorescent protein (mNG)-tagged versions of the proteins are stably expressed as intact fusions, as checked by fluorescence microscopy and Western blotting. We were not able to inmmunodetect Mmm1- and Mdm34-mNG, probably due to the sensitivity of the anti-mNG antibody used. Instead, the correct C-terminal fusion of the mNG tag in the genomic loci was checked by colony PCR. The *Escherichia coli* DH5α F’(K12Δ(*lacZYA*-*argF*)U169 *deoR supE44 thi-1 recA1 endA1 hsdR17 gyrA96 relA1* (φ*80lacZ*Δ*M15*)F’) was the routine strain used for general molecular biology protocols. For Gateway cloning, we used the *S. cerevisiae* Advanced Gateway Destination Vectors collection from Susan Lindquist (Addgene Kit # 1000000011) [33] that was provided in a *ccdB* resistant *E. coli* strain.

YPD [1% (*w/v*) yeast extract, 2% (*w/v*) peptone and 2% (*w/v*) glucose] broth or agar was used as a general non-selective medium for growing yeast cells. Synthetic dextrose complete medium (SD) consisted of 0.17% yeast nitrogen base without amino acids, 0.5% ammonium sulfate, 2% glucose, and the appropriate amino acids and nucleic acid bases supplements, was used for plasmid selection and maintenance. SG and SR were SD with 2% galactose or 1.5% raffinose, respectively, instead of glucose. *E. coli* was cultured using the Luria Bertani (LB) medium (1% tryptone, 0.5% yeast extract, and 0.5% NaCl), supplemented for plasmid selection with 100 μg/mL ampicillin or 50 μg/mL kanamycin. *GAL1*-driven protein induction in liquid medium was performed by growing *S. cerevisiae* cells in SR for 18 h and then refreshing them in SG to an OD_600_ of 0.3, and cultured for additional 5 h. Spot growth assays on agar were performed as described [34].

### 2.2. Plasmid Construction

General molecular biology techniques were performed by standard methods. Yeast transformation was achieved by the standard lithium acetate protocol. All plasmids and oligonucleotides used in this work are listed in Supplementary Tables S2 and S3 respectively.

For restriction enzyme cloning, PCR products were subcloned into the pGEM-T vector system and the insert was then subcloned into a yeast expression vector. For Gateway cloning (ThermoFisher, Waltham, MA, USA), primers containing the *attB* sequences were designed as indicated by the user guide. Inserts were amplified and later introduced into pDONR221 (ThermoFisher) using the BP clonase II (ThermoFisher) to obtain the entry plasmid. Destination vectors were chosen from the *S. cerevisiae* Advanced Gateway Destination Vectors collection (Addgene Kit #1000000011) [33]. Sequenced inserts from entry plasmids were subcloned into the chosen destination vectors using the LR clonase II (ThermoFisher) to generate the yeast expression plasmids. As destination vectors bearing the ccdB gene are not suitable for *E. coli* DH5α transformation, the polylinker region of the pEG(KG) vector was cloned to remove the *ccdB* gene and obtain a suitable control plasmid for the yeast expression experiments.

Human cDNA of *TLR4* and the adaptors *MyD88*, *TIRAP*, *TRIF*, and *TRAM* was used for insert amplification. Point mutations or insertions were generated via the site-directed mutagenesis method using the *Pfu*I Turbo DNA polymerase (Agilent Technologies, Santa Clara, CA, USA). Primers were designed following the guidelines on the QuikChange kit (Agilent) instructions and contained the desired mutation.

### 2.3. Microscopy Techniques, Image Processing, and Statistical Analysis

For in vivo fluorescence microscopy (GFP, EGFP, or mCherry), cultures subjected to *GAL1* induction were concentrated by centrifugation at 2500 rpm for 1 min. Cells were examined in Eclipse TE2000U microscope (Nikon, Tokyo, Japan) and digital images were acquired with an Orca C4742-95-12ER charge-coupled-device camera (Hamamatsu Photonics, Hamamatsu City, Japan) and processed by HC Image (Hamamatsu). To monitor vacuolar morphology and endocytosis, staining with FM4-64 was performed as described [35]. For calcofluor white stain, exponentially growing cells were harvested at 4500 rpm for 2 min and then washed with PBS twice. Cells were stained with 5 µg/mL calcofluor-white (Sigma-Aldrich, St. Louis, MO, USA) for 10 min protected from light. Finally, they were collected and washed four times and observed at the fluorescence microscope. For confocal microscopy, cells were treated as previously described [36]. Confocal images were acquired using a Zeiss LSM 510 confocal microscope (Carl Zeiss, Oberkochen, Germany), and an Olympus Ix83 inverted microscope, coupled to Olympus FV1200 confocal system (Shinjuku, Tokyo, Japan). Images taken with the first one were processed using SlideBook6 (3i Intelligent imaging, Denver, CO, USA), and FiJi (ImageJ; https://imagej.net/software/fiji/) [37] and Adobe Photoshop CS6 (Mountain View, CA, USA) were used in the second case. As a quantitative approach for co-localization of green and red channels, Pearson correlation was calculated using the JACoP plugin for ImageJ [38].

All data sets were tested for normality using the Shapiro-Wilk test. When a normal distribution was confirmed a One-Way ANOVA test with a Bonferroni correction was used for statistical comparison of multiple data sets and Students *t*-test for two-sample comparison. For data sets that did not show normality, a Kruskal-Wallis test was applied, with Dunn’s correction. In all cases a significance level (alpha) of 0.05 was selected.

### 2.4. Immunodetection by Western Blotting

Standard procedures were used for yeast cell growth, collection, breakage, protein separation by SDS-PAGE, and transfer to nitrocellulose membranes, as previously described [39]. The protein lysis buffer composition is as follows: 50 mM Tris-HCl pH 7.5, 10% glycerol, 1% Triton X-100, 0.1% SDS, 150 mM NaCl, and 5 mM ethylenediaminetetraacetic acid (EDTA). Shortly before use was supplemented with phenylmethylsulfonyl fluoride (PMSF) up to 1 mM, dithiothreitol (DTT) up to 10 mM, and protease inhibitor cocktail (Roche/Sigma-Aldrich, St. Louis, MO, USA).

GFP and EGFP fusion proteins were detected using a monoclonal anti-GFP antibody (Living Colors, JL-8, Takara Bio, Kusatsu, Japan; 1:1000); mCherry fusions were identified with a polyclonal anti-DsRed antibody (Living Colors, 1:1000); GST-tagged proteins were recognized by an anti-GST polyclonal antibody (z5, Santa Cruz Biotechnology, Dallas, TX, USA; 1:1000); and mNG fusions were detected with anti-mNeonGreen antibody (32F6, Chromotek, Planegg-Martinsried, Germany; 1:500). A yeast-specific polyclonal anti-Glucose-6-Phosphate Dehydrogenase (G6PDH; Sigma-Aldrich, St. Louis, MO, USA) diluted 1:50,000 was included as a loading control. In all cases, primary antibodies were detected using IRDye-680 or -800 anti-rabbit or anti-mouse goat polyclonal antibodies (Li-Cor Biosciences, Lincoln, NE, USA), all of them diluted 1:5000, with an Odyssey Infrared Imaging System (Li-Cor Biosciences, Lincoln, NE, USA).

### 2.5. Protein Co-Purification Assays

Cultures subjected to *GAL1* induction were harvested and lysed with 300 µL of ice-cold lysis buffer [10% (*v/v*) glycerol, 50 mM Tris/HCl pH 7.5, 0.1% (*v/v*) NP40, 150 mM NaCl, 5 mM EDTA pH 8, 50 mM NaF, 5 mM sodium pyrophosphate, 50 mM β-glycerol phosphate, and 1 mM sodium orthovanadate], and they were clarified by centrifugation at 13,000 rpm for 10 min at 4 °C. Shortly before use, the lysis buffer was supplemented with PMSF up to 1 mM and protease inhibitor cocktail (Roche/Sigma-Aldrich). Once protein extracts were diluted to the same concentrations, 10 μL were saved as input samples. To equilibrate the beads, three washes with ice-cold lysis buffer were performed in the case of GST pull-down experiments (10 mM Tris-HCl pH 7.5, 150 mM NaCl, and 0.5 mM EDTA pH 8). Then, 50 µL of Glutathione Sepharose beads (diluted at 50%) and 25 µL of GFP-TrapA slurry were added to 150 µL of the resting extracts. To ensure proper slurry-sample mixing, the final volume was increased up to 600 μL with lysis buffer in either case and tubes were tumbled end-over-end overnight at 4 °C. Then, samples were centrifuged at 4 °C, the supernatant was discarded, and pellets were washed with ice-cold lysis buffer in the case of GST pull-down and with Dilution buffer in the GFP-TrapA co-immunoprecipitation. Washed pellets were treated with 2× loading SDS-PAGE buffer [125 mM Tris/HCl pH 6.8, 5% SDS, 25% (*v/v*) glycerol, 0.2 M DTT, and 0.1% bromophenol blue] and boiled for 5 min to denature and elute the proteins from the slurry. These samples, together with the input samples, were loaded on a polyacrylamide gel to be visualized by Western blotting as described.

## 3. Results

### 3.1. The TIRAP Adaptor Tightly Localizes to the Yeast PM

Among TIR-domain containing adaptors involved in innate immunity signaling, TIRAP plays a preeminent role. Its N-terminal extension has been reported to be responsible for its localization to cellular membranes, notably the PM, as a consequence of its interaction with PtdIns(4,5)P_2_ [7]. *S. cerevisiae* is a model for molecular studies on phosphoinositide-dependent signaling, and like higher eukaryotic cells, its PM is enriched in PtdIns(4,5)P_2_ [40,41]. To track in vivo behavior of TIRAP in yeast, we developed a system for the heterologous expression, from *GAL1* promoter, of human cDNA encoding TIRAP fused to mCherry at both the N- and the C-terminus. This promoter is dependent on the carbon source, being tightly repressed in glucose- and highly activated in galactose-based growth media. Expression of these protein fusions in extracts from cells grown in galactose-based media was checked by immunobloting, and did not cause growth defects on yeast cells (Supplementary Figure S1). We have recently described that TIR domains that bear NAD^+^ hydrolase activity are severely toxic in yeast [42], so tolerance to high levels of expression is consistent with the fact that such enzymatic activity has not been reported in this particular human innate immune TLR adaptor.

**Figure 1:**
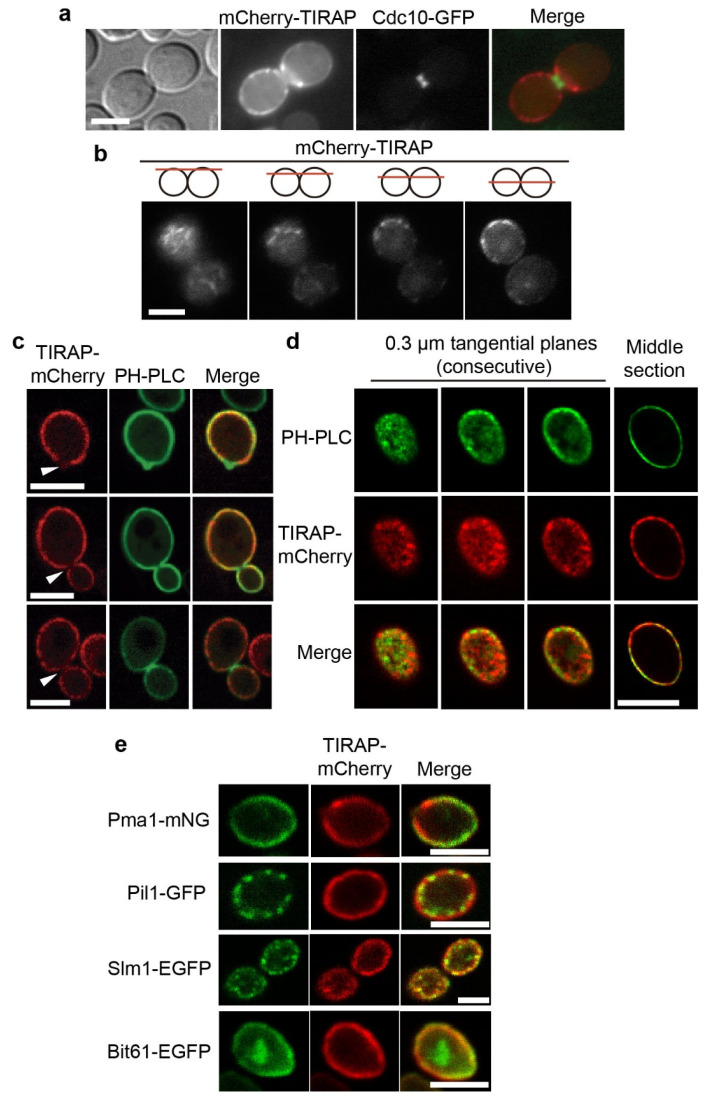
Human TIRAP localizes to the PM in yeast cells. (**a**) Differential interference contrast and fluorescence microscopy of YPH499 yeast cells expressing mCherry-*TIRAP* from pYES2 (red) and *CDC10*-GFP from pLA10H (green). (**b**) Four different focuses of the same cell expressing mCherry-*TIRAP* from pYES2. Focus on the cell surface allows visualization of short filaments (leftmost image) that in the middle plane often appear as spots (right). (**c**) Selected laser confocal microscopy of YPH499 yeast cells expressing *TIRAP*-mCherry from pYES3 (red) and GFP-PH(*PLCδ*) (PH-PLC) from pRS426, a PtdIns(4,5)P_2_ marker (green). In contrast to PH-PLC, TIRAP-mCherry is missing from the emerging bud area (upper panel), as well as from the bud neck both in small- (middle panel) and large-budded (lower panel) cells (arrowheads). The middle panel shows a typical cell in which the TIRAP-mCherry signal is fainter in the emerging bud than in the mother cell. (**d**) Different confocal planes of a representative cell as in (**c**), showing a differential PM pattern for the green (PH-PLC) and red (TIRAP-mCherry) channels. (**e**) Laser confocal microscopy of yeast cells expressing *TIRAP*-mCherry from pYES3 (red) and displaying different membrane compartments tagged in green: the MCP (Membrane Compartment containing Pma1) component Pma1-mNG (from EVY3 yeast strain), the MCC (Membrane Compartment containing Can1) component Pil1-GFP (from TWY110 yeast strain), the MCC/MCT (Membrane Compartment containing TORC2) component Slm1-EGFP (from a pAG413GPD derived plasmid), and the MCT component Bit61-EGFP (from a pAG413GPD derived plasmid). Scale bars correspond to 5 μm.

As expected, both mCherry-TIRAP (Figure 1a,b) and TIRAP-mCherry (Figure 1c–e) localized to the yeast PM. The C-terminal fusions clearly decorated the PM in a homogeneous fashion (Figure 1a,b). However, the N-terminal fusions did not show such a continuous PM localization pattern. This is likely due to the presence of a bulky tag close to the N-terminal polybasic stretch, where PM-localization signals are located [7]. mCherry-TIRAP tended to form peripheral clusters rather than a continuous PM pattern, and often concentrated near the bud region, adjacent but excluded from the septin ring, as determined by Cdc10-GFP septin localization (Figure 1a). Focal analyses revealed that mCherry-TIRAP clusters often had the shape of short filaments (Figure 1b).

To test whether TIRAP localization matches that of PtdIns(4,5)P_2_ in the PM, we used confocal microscopy and the PtdIns(4,5)P_2_-specific reporter GFP-PH(PLCδ). By taking confocal sections in the middle plane of the cells, we found that, while the PtdIns(4,5)P_2_ marker was enriched at growing buds and bud necks, as expected [43], TIRAP-mCherry was excluded from bud necks. Peculiarly, TIRAP-mCherry was more abundant in the mother cell PM than in emerging and growing buds, although large buds had a TIRAP-mCherry PM signal at the daughter cell, equivalent to that of the mother (Figure 1c). This observation likely indicates that the septin ring, which tightly assembles at the bud neck, acts as a diffusion barrier for TIRAP-mCherry. Confocal microscopy revealed that the TIRAP-mCherry signal did not exactly match the PtdIns(4,5)P_2_ marker (Figure 1d), suggesting that environmental determinants at the PM other than PtdIns(4,5)P_2_ influence its precise localization.

The yeast PM is patched with different microdomains, like Membrane Compartments rich in Can1, Pma1, or TORC2 (respectively named MCCs, MCPs, and MCTs) [44]. We observed localization of TIRAP-mCherry together with the localization of the MCP marker Pma1-mNG, the MCC marker Pil1-GFP, the MCT marker Bit61-EGFP and the PtdIns(4,5)P_2_-binding protein Slm1-EGFP, which shifts between MCCs and MCTs [45]. As expected, whereas Pil1-GFP and Slm1-EGFP were localized in well-defined patches across the membrane, Pma1-mNG had a more homogeneous pattern. Bit61-EGFP, however, did not mark in our hands discrete spots at the PM consistent with MCTs, but displayed a rather continuous signal, enriched at growth sites, like the nascent bud (Figure 1e and Figure S2). TIRAP-mCherry partially overlapped at the PM with all four markers, showing the best co-localization score with Bit61-EGFP (Pearson coefficient 0.801 ± 0.018), followed by Pma1 (0.768 ± 0.023) and Slm1-EGFP (0.732 ± 0.035). The lowest co-occurrence was with the MCC marker Pil1-GFP (0.592 ± 0.034) (Figure 1e and Figure S2). Consistently, TIRAP signal was not particularly enriched at MCCs, although it seemed often concurrent with certain Slm1-EGFP spots (Figure 1e and Figure S2b). Likewise, unlike Bit61-EGFP, TIRAP-mCherry was not enriched in growing, PtdIns(4,5)P_2_-rich areas (Figure S2c), indicating that its binding to the PM is not linked to endo/exocytic events. It is noteworthy that neither GFP-PH(PLCδ) nor Pma1-mNG were excluded from the septin-delimited bud neck, while TIRAP-mCherry was. This may suggest that TIRAP forms highly ordered structures that cannot diffuse through the septin barrier to bind PtdIns(4,5)P_2_, which is enriched in the bud neck area.

### 3.2. Lysine Residues in the Polybasic Motif of TIRAP Determine PM Localization in Yeast

**Figure 2:**
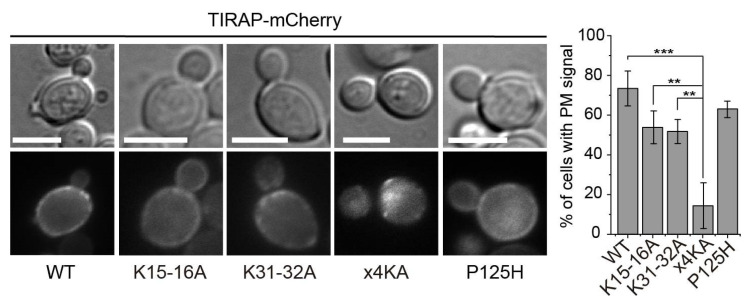
The TIRAP polybasic region targets it to the yeast PM. Differential interference contrast and fluorescence microscopy of YPH499 yeast cells expressing WT TIRAP-mCherry and the indicated mutants from pYES3 derivative plasmids (left). Graph displaying the percentage of cells showing PM mCherry fluorescent signal (right). Data correspond to means ± standard deviation of three independent transformants (*n* > 100). Normality was checked by the Shapiro–Wilk test. One-way ANOVA statistical comparison produced a *p*-value < 0.001 (***) for ×4KA vs. WT [*p* = 4.8 × 10^−5^], and a *p* < 0.01 (**) vs. K15-16A (*p* = 0.00147) and K31-32A (*p* = 0.00222). Scale bars correspond to 5 μm.

The results above prove that the TIRAP inherent PM-localization determinants drive the protein to the PM in the absence of additional components of the higher eukaryotic TLR signaling pathway, so the yeast model is suitable to study TIRAP interactions with the PM in vivo. We used site-directed mutagenesis to study the involvement of Lys residues in the polybasic region defined as a PtdIns(4,5)P_2_-binding motif (PBM). For this purpose, we independently changed to Ala two Lys pairs, namely Lys15-Lys16 (K15-16A) and Lys31-Lys32 (K31-32A). In addition, we produced the corresponding quadruple mutant (×4KA), lacking all positively charged amino acids reported to configure the PBM of TIRAP [7,8]. The presence of all mutants was verified by immunoblotting and, like WT TIRAP, their production did not affect yeast growth (Figure S3a–c). As shown in Figure 2, both double mutations slightly reduced the percentage of yeast cells with their PM decorated with TIRAP-mCherry, whereas the quadruple substitution abrogated TIRAP-mCherry PM localization. The immunoblot revealed that the ×4KA mutant showed a mobility pattern different to that of the WT and the double mutants, missing the bands of lower mobility (Figure S3b), suggesting that impairing TIRAP membrane tethering affects its stability or its ability to undergo posttranslational modifications. To test whether PM localization was dependent on the ability of TIRAP to homopolymerize via TIR-TIR interactions, we generated the P125H mutant, with a canonic substitution in a Pro residue at the BB loop which is predicted to impair self-interaction [10]. TIRAP^P125H^-mCherry still showed a predominant PM localization (Figure 2). These results support previous evidence that TIRAP PM recognition relies on the polybasic domain of its non-TIR N-terminal extension and is independent of its TIR domain.

### 3.3. The TRAM Adaptor Forms Long PM-Tethered Filaments When Expressed in Yeast

**Figure 3:**
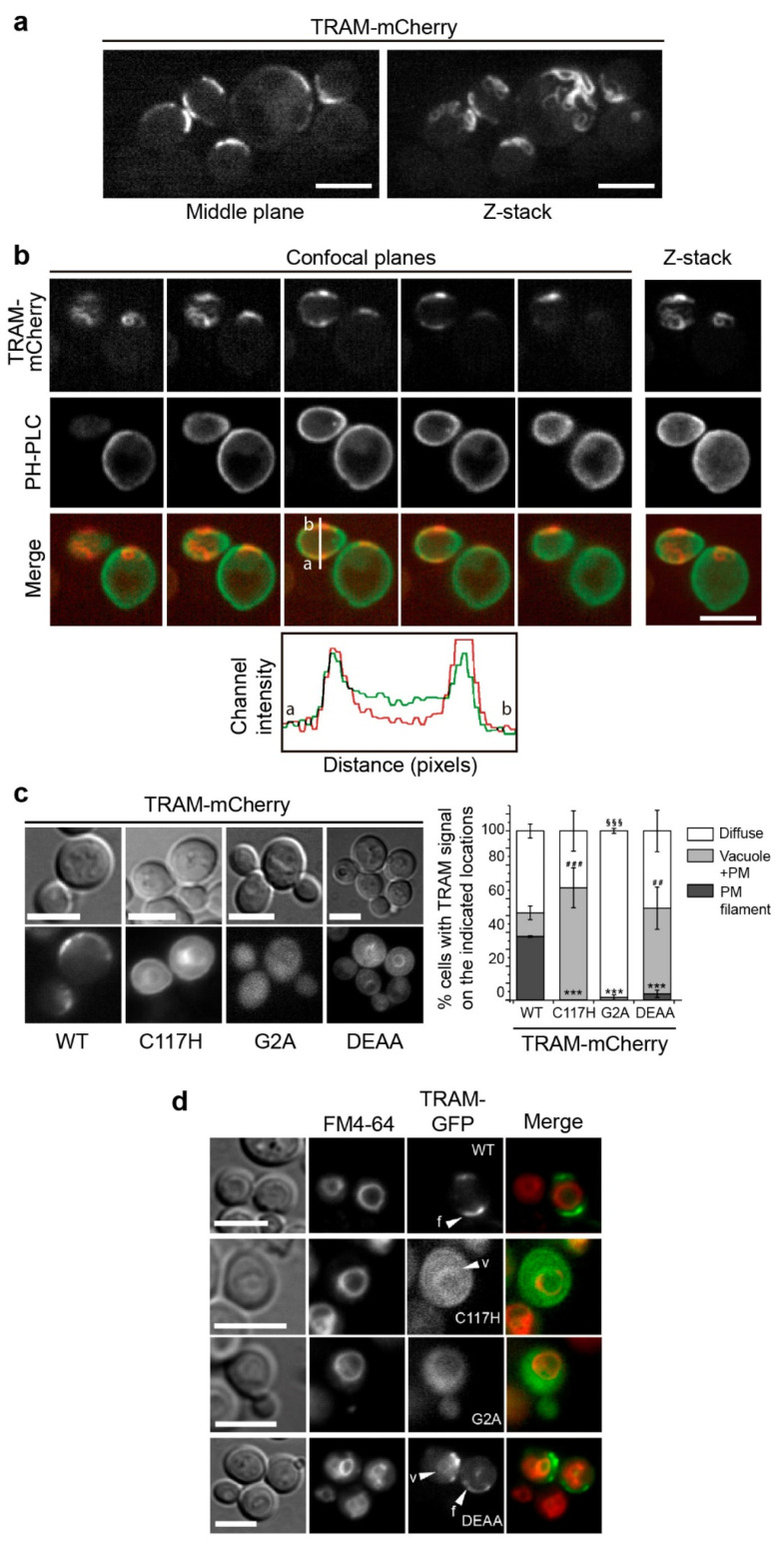
The TRAM adaptor forms long PM-tethered filaments when expressed in yeast. Confocal images of YPH499 yeast cells expressing TRAM-mCherry alone from a pYES3 derivative plasmid (**a**) or co-expressing it with the PtdIns(4,5)P_2_ marker GFP-PH(PLCδ) (PH-PLC) from pRS426 (**b**). In (**a**) Z-stacks are the projection of 19 planes (0.25 μm per step). In (**b**), five representative confocal planes and the corresponding Z-stack are shown. The histogram depicts arbitrary units of fluorescence intensity vs. distance in pixels from the line distance *a* to *b* marked in the middle plane, as indicated. (**c**) Differential interference contrast and fluorescence microscopy of YPH499 yeast cells bearing WT TRAM-mCherry and the indicated mutants, expressed from pYES3 derivative plasmids (left); and a graph displaying the percentage of yeast cells showing TRAM-mCherry signal on either PM filaments, vacuoles, or diffuse in the cytosol (right). Data correspond to means ± standard deviation of three independent transformants (*n* > 100). Normality was checked by the Shapiro–Wilk test. One-way ANOVA statistical comparison of filament data led a *p*-value < 0.001 (***) for the WT vs. the other three mutants: C117H [*p* = 8.8 × 10^−10^], G2A [*p* = 8.8 × 10^−10^], and DEAA [*p* = 2 × 10^−9^]. One-way ANOVA statistical comparison of vacuole data led a *p*-value < 0.001 (###) for WT vs. C117H [*p* = 5.3 × 10^−4^], and a *p* = 0.00575 (##) for WT vs. DEAA. One-way ANOVA statistical comparison of cytosol data led a *p*-value < 0.001 (§§§) for WT vs. G2A [*p* = 6.8 × 10^−4^]. (**d**) Differential interference contrast and fluorescence microscopy of YPH499 yeast cells bearing TRAM-GFP and the indicated mutants, from YCpLG plasmid derivatives, after 1h treatment with the FM4-64 vital dye. White arrows indicate TRAM on vacuolar membranes (v) or filaments (f). All scale bars correspond to 5 μm.

TRAM is an alternative adaptor in TLR signaling, connecting TLR4 and TRIF. It has an N-terminal bipartite sorting signal, comprising a myristoylation signal and an endosomal localization motif, which tethers TRAM to the PM and to endosomal membranes in the mammalian cell [18]. To preserve these properties, C-terminal fusions of TRAM to fluorescent proteins were studied in the yeast cell. As shown in Figure 3, TRAM formed long curly filaments along the yeast PM in a substantial proportion of cells (Figure 3a). Confocal imaging of yeast cells co-producing TRAM-mCherry and GFP-PH(PLCδ), the PtdIns(4,5)P_2_ marker, showed both signals merging at the middle plane (Figure 3b), indicating that TRAM structures overlapped the PM marker, probably due to TRAM N-terminal myristoylation signal. Since TRAM shapes occasionally form closed circles associated with the periphery of the cell that could remind of bud scars, we performed a calcofluor white stain, which ruled out that they were linked (Figure S4).

To gain insight into the features involved in this localization pattern, we generated a series of TRAM mutations: (i) a change of the Cys117 residue in a conserved region of the TIR domain BB loop, C117H [10]; (ii) a G2A substitution, yielding an inactive myristoylation signal [46]; and (iii) the substitution for Ala of two acidic residues previously identified as key for proper TRAM endosomal location [46], (D91A E92A, designated as DEAA). The production of none of these mutants altered yeast growth even if they were efficiently produced upon induction in the yeast cell, as determined by immunoblotting (Figure S3d–g).

Interestingly, the BB loop mutant TRAM^C117H^-GFP, which presumably fails to self-interact, did not form filaments. Instead, it homogeneously decorated the PM, as well as inner membranes (Figure 3c). By co-staining of the endocytic compartments with the fluorescent marker FM4-64, we determined that inner membranes corresponded to the yeast vacuole (Figure 3d), indicating that TRAM is either not properly sorted to the PM or readily endocytosed when its ability to form filaments is impaired. On the other hand, when deprived of its N-terminal myristoylation signal, the TRAM^G2A^-GFP mutant appeared diffuse in the cytosol, where it failed to develop filamentous structures (Figure 3c,d). Although we cannot discard that altering the myristoylation signal may affect TRAM structure, our results suggest that efficient PM targeting is a prerequisite for TRAM to form filaments. Finally, the TRAM^DDAA^-GFP mutant, like the C117H point mutant, was mostly found in the PM and endocytic membranes, although some cells (3.6% ± 2.2 s.d. *n* > 100) were still able to generate the typical PM-attached filaments (Figure 3c,d). This points out that these two acidic residues are important but not critical for TRAM filament formation in the yeast model.

### 3.4. Co-Expression of TLR4 TIR Domain in Yeast Leads to Nucleation of TIRAP and TRAM In Vivo

Next, we aimed to test whether interactions of the human TLR4 TIR domain with either TIRAP or TRAM could be reproduced in the yeast cell. We chose to express only the cytosolic C-terminal region (amino acids 657–839), containing the TIR domain, of human TLR4, as we predicted that the extracellular domains of full-length TLR4 would interfere with the yeast cell wall. To mimic the homodimerization of the receptor, we expressed it as an N-terminal fusion to GST, as this protein tag is known to dimerize. Also, a construct was developed with a myristoylation (myr) signal at the N-terminus of the GST tag, in order to target the construct to the PM, thus mimicking the actual location of the TLR4 TIR domain attached to the inner layer of the PM. Like in the case of TIRAP and TRAM, the expressed GST-TLR4(TIR) fusions, when produced in yeast cells, did not alter their growth (Figure S5).

**Figure 4:**
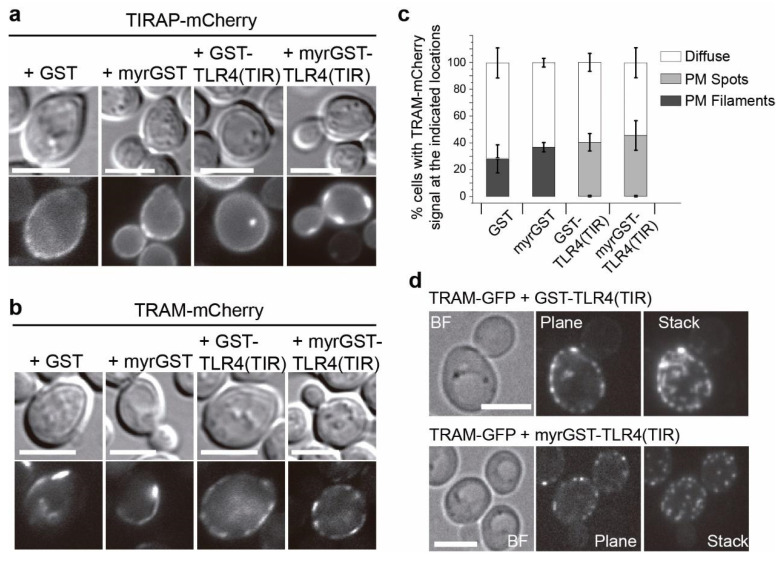
TLR4(TIR) relocates TIRAP and impairs TRAM filament formation. Differential interference contrast and fluorescence microscopy of YPH499 yeast cells co-transformed with pEG(KG) derivatives expressing bare [GST] or myristoylated GST [myrGST], bare [GST-TLR4-(TIR)] or myristoylated [myrGST-TLR4(TIR)] GST fusion to TLR4(TIR) together with TIRAP-mCherry from pYES3 (**a**) or TRAM-mCherry from pYES3 (**b**). Cultures were grown on SR lacking not only uracil and tryptophan but also leucine to ensure maximum plasmid copy number in pEG(KG)-derivatives. (**c**) Graph displaying the percentage of cells showing TRAM-mCherry fluorescent signal predominantly as PM filaments (black), or as PM puncta (gray). Data correspond to means ± standard deviation of three independent transformants (*n* > 100). (**d**) Bright-field (BF) and confocal microscopy of YPH499 yeast cells co-transformed with TRAM-GFP from YCpLG and either GST-TLR4(TIR) or myr-GST-TLR4(TIR) from pEG(KG) derivative plasmids. Stacks are the Z projection of 15 planes [GST-TLR4(TIR)] or 14 planes [myrGST-TLR4(TIR)] of 0.3 μm per step. Scale bars correspond to 5 μm.

The plasmids coding for GST-TLR4(TIR) fusions were co-transformed with those coding for fluorescent protein-tagged versions of either TIRAP or TRAM and the localization pattern of both adaptors was analyzed by fluorescence microscopy. TIRAP-mCherry, besides its typical yeast PM localization, was also found in cytosolic spots when non-myristoylated GST-TLR4(TIR) was co-expressed, but not in control cells producing either GST or myr-GST alone. Remarkably, in cells with the PM-targeted construct myr-GST-TLR4(TIR), TIRAP-mCherry was driven to PM-bound large foci (Figure 4a). This suggests that the TLR4 TIR domain is able to nucleate TIRAP in the yeast heterologous model.

TLR4(TIR) co-production with TRAM-mCherry also altered the characteristic TRAM-mCherry PM-bound filaments. Presence of either myristoylated or non-myristoylated GST-TLR4(TIR) constructs led to a TRAM-mCherry localization pattern consistent of PM puncta, rather than filaments, in virtually all cells (Figure 4b,c). Further confocal imaging revealed that those spots were shortened filaments when accompanied by the presence of GST-TLR4(TIR), and discrete dots when myrGST-TLR4(TIR) was present (Figure 4d). The observation that co-expression of TLR4(TIR) drastically interfered with the formation of TRAM filaments likely reflects heterotypic TIR-TIR interactions in vivo.

### 3.5. MyD88 and TRIF Nucleate at Cytoplasmic Spots, a Pattern Unaltered by Key TIR Mutations

**Figure 5:**
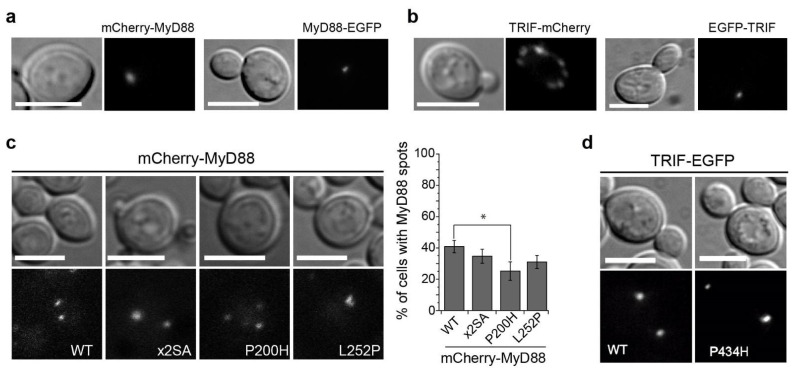
Point mutants in the TIR domain of MyD88 and TRIF do not alter their cytoplasmic spot localization. (**a**) Differential interference contrast and fluorescence microscopy of YPH499 yeast cells expressing either mCherry-MyD88 or MyD88-EGFP from plasmid pYES3 and pAG425GAL, respectively. (**b**) TRIF-mCherry or EGFP-TRIF from plasmids pYES3 and pAG425GAL, respectively. (**c**) Differential interference contrast and fluorescence microscopy of YPH499 yeast cells bearing mCherry-MyD88 WT and the indicated mutants, from pYES3 derivative plasmids (left) and a graph displaying the percentage of yeast cells showing MyD88 spots (right). Data correspond to means ± standard deviation of three independent transformants (*n* > 100). Normality was checked by the Shapiro–Wilk test. One-way ANOVA statistical comparison retrieved a *p*-value = 0.021 (*) for WT vs. P200H mutant. (**d**) Differential interference contrast and fluorescence microscopy of YPH499 yeast cells bearing EGFP-TRIF WT and the P434H mutant from pAG425GAL-EGFP derivative plasmids. Scale bars correspond to 5 μm.

Next, we expressed fusions of TIR domain-containing adaptors MyD88 and TRIF to fluorescent proteins. Like TRAM and TIRAP, they were properly expressed in yeast and lacked toxicity (Figure S1). N- and C-terminal fusions were developed for both MyD88 and TRIF. At the microscope, both fluorescent protein fusions appeared as cytoplasmic puncta. Both mCherry-MyD88 and MyD88-EGFP were usually in 1 to 4 cytosolic spots per cell, although a single spot was the most common situation (Figure 5a). Regarding TRIF, N-terminal tagged EGFP-TRIF showed 1 or 2 dots, whereas C-terminally tagged TRIF-mCherry typically displayed more than 5 puncta per cell (Figure 5b). When EGFP-TRIF and TRIF-mCherry were co-produced, they co-localized at 1 or 2 spots per cell (Figure S6), indicating that at least some of the TRIF-decorated spots were common for both fusions.

To test whether MyD88 intermolecular TIR-TIR interactions were necessary for the assembly of the discrete cytoplasmic spots above described, we targeted by site-directed mutagenesis residues within the MyD88 TIR domain that had been reported to mediate homotypic interaction. First, we generated a Ser to Ala double mutant (S242A-S244A, designated ×2SA), with substitutions of two residues presumably involved in regulation of myddosome assembly by phosphorylation [47]. Second, we introduced a Pro to His substitution at the BB loop (MyD88^P200H^), equivalent to that in TIRAP^P125H^ [9; 10] that impairs TIR-TIR interactions. And third, we reproduced a purported oncogenic mutation which seems to result in stronger interacting properties (MyD88^L252P^) [48,49,50]. As verified by immunoblotting, all MyD88 mutants were produced, although the band of L252P mutant was fainter, and, like the WT, presence of none of the mutants caused toxicity in yeast cells (Figure S3h,i). Fluorescence microscopy showed that all mCherry-MyD88 mutants typically displayed 1 to 4 mCherry-MyD88 cytosolic spots, although in a lower proportion of cells as compared to the WT, which was statistically significant for MyD88^P200H^ (Figure 5c). This result probably reflects the impaired self-interacting properties of this mutant, but since cytoplasmic spots could still be formed, we can conclude from this experiment that subcellular localization of MyD88 in the yeast cell is independent of TIR-TIR interactions. We also introduced a single point mutation in the TIR BB loop of TRIF by changing the Pro434 to His (TRIF^P434H^), the equivalent residue in TRIF structure to Pro200 in MyD88. Like WT TRIF, the mutant was localized as 1–2 cytoplasmic spots per cell (Figure 5d). Thus, like in the case of MyD88, impairing the TIR domain self-interacting properties in TRIF did not alter its localization pattern.

### 3.6. Human MyD88 Localizes to the Yeast ERMES

**Figure 6:**
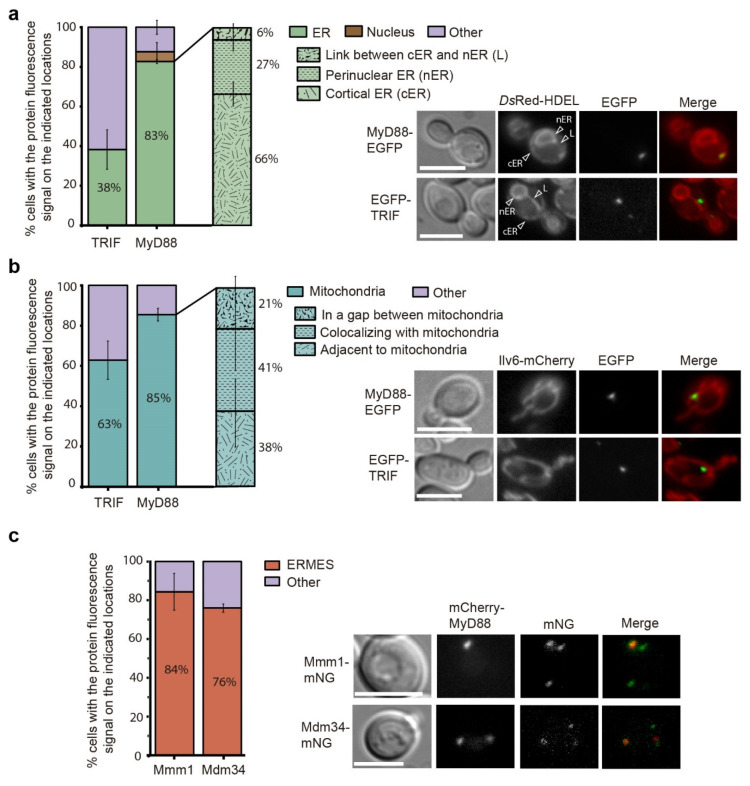
MyD88 is associated to ER-mitochondrial encounter structure (ERMES). (**a**) Differential interference contrast and fluorescence microscopy of yeast cells expressing MyD88-EGFP or EGFP-TRIF in the VHY87 strain, which bears the endoplasmic reticulum marker DsRed-HDEL (right), and graph (left) displaying the percentage of yeast cells showing MyD88-EGFP and EGFP-TRIF spots colocalizing with the indicated endoplasmic reticulum (ER) locations: cortical ER (cER), perinuclear ER (nER) or the link between both (L), as indicated by arrowheads in the image. Data correspond to means ± standard deviation of three independent transformants (*n* > 90). (**b**) Differential interference contrast and fluorescence microscopy of yeast cells co-expressing MyD88-EGFP or EGFP-TRIF with the mitochondrial marker Ilv6-mCherry on the BY4741*trp1*Δ strain (right), and graph displaying the percentage of yeast cells showing the indicated relative positions between MyD88-EGFP and EGFP-TRIF, and the mitochondria (left). Data correspond to means ± standard deviation of three independent transformants (*n* > 90). (**c**) Differential interference contrast and fluorescence microscopy of yeast cells expressing mCherry-MyD88 in the EVY4 and EVY5 strains, that bear the ERMES components Mdm34 and Mmm1, respectively, tagged with mNeonGreen (right). Graph displaying the percentage of cells showing colocalization between MyD88 and each marker (left). Data correspond to means ± standard deviation of three independent transformants (*n* > 150).

Next, we assayed whether MyD88 and TRIF spots were associated with cytoplasmic membranes. We first tested their localization with mCherry-P4C(SidC), a mCherry fusion of the PtdIns4P binding region (P4C) from the *Legionella pneumophila* effector SidC [51,52,53] which marks Golgi, but neither EGFP-MyD88 nor EGFP-TRIF colocalized with this marker (Figure S7). Then, we used fluorescent markers for the endoplasmic reticulum (ER) and mitochondria. As shown in Figure 6a,b, MyD88-EGFP overlapped with the ER marker DsRed-HDEL in 82.68% ± 1.06 s.d. of the cells (*n* > 90), and it was often found co-localizing or adjacent to yeast mitochondria, as detected by co-localization with the mitochondrial marker Ilv6-mCherry (85.47% ± 3.17, *n* > 90). In contrast, only a few cells displayed EGFP-TRIF spots merged with ER membranes (38.28% ± 9.98, *n* > 90) and EGFP-TRIF was also found less frequently adjacent to mitochondria (62.82% ± 9.51, *n* > 90). Given the proximity of MyD88-EGFP to both organelles, we wondered whether it could be located at the ER-mitochondrial encounter structure (ERMES) junction [54]. In order to check this hypothesis, we constructed strains bearing a mNeonGreen tag in two components of the ERMES complex: Mdm34, found in the outer mitochondrial membrane (OMM), and Mmm1, an ER membrane protein [55]. Interestingly, in most cells mCherry-MyD88 spots precisely co-localized with both ERMES components Mmm1-mNG (84.34% ± 9.48, *n* > 150) and Mdm34-mNG (75.94% ± 2.09, *n* > 150) (Figure 6c). These results clearly indicate that, when expressed in yeast, MyD88 has affinity for mitochondrion-ER junctions.

Heterologous production of proteins in yeast cells often results in the formation of unfolded protein aggregates, visible in microscopic images as clusters, inclusions or spots. Such structures are often marked by Hsp104, a chaperone involved in the disassembly of heat shock-induced stress granules (HS-SGs). To add further evidence of MyD88 location in the yeast model, we constructed a strain in which Hsp104 was labeled with mNeonGreen. Under optimal growth conditions, Hsp104-mNG is homogeneously distributed in the cytosol and enriched in the nucleus. We clearly saw that mCherry-MyD88 overexpression did not modify this pattern (Figure S8). However, heat shock at 42 °C for 30 min resulted in the appearance of several Hsp104-mNG-labeled stress granules per cell. In this situation, mCherry-MyD88 still formed 1–4 aggregates per cell, and they colocalized with particular stress granules. It has been recently shown that some HS-SGs associate with ERMES [56], so this result is in agreement with our previous findings that mCherry-MyD88 is located at these junctions.

### 3.7. Co-Expression of TIRAP and MyD88 Leads to TIR-Dependent Mutual Recruitment

**Figure 7:**
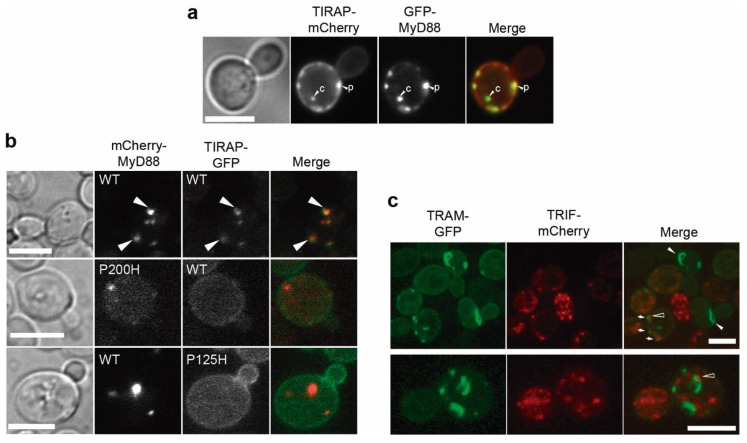
Co-expression of human TIR adaptor pairs. (**a**) Bright field and fluorescence microscopy of YPH499 yeast cells co-transformed with pYES3-*TIRAP*-mCherry and pYES2-GFP-*MyD88*. White arrows indicate co-localization events on cytosolic spots “c” or PM patches “*p*”. (**b**) Bright field and fluorescence microscopy of YPH499 yeast cells co-expressing mCherry-MyD88 from pYES3 and TIRAP-GFP from YCpLG, either WT or the indicated mutants. Co-localization occurs only in the WT and is marked with arrowheads. (**c**) Laser confocal microscopy of YPH499 cells expressing TRAM-GFP from YCpLG and TRIF-mCherry from pYES3. Images are maximum Z projection stacks of 12 (upper panel) and 13 (lower panel) confocal planes. Small arrows point TRAM-GFP non-filamentous patches, white arrowheads mark long TRAM-GFP filaments in cells lacking TRIF-mCherry signal, and hollow arrowheads indicate minor TRIF-mCherry spots that were located adjacent to TRAM-GFP spots. Scale bars represent 5 μm.

Next, we used the yeast model to study heterotypic TIR-TIR interactions between adaptors when co-expressed in yeast. GFP-MyD88 and TIRAP-mCherry clearly colocalized, since both proteins merged at either PM patches or MyD88 cytoplasmic spots (Figure 7a). Importantly, this result recapitulates TIRAP-MyD88 interaction in the yeast cell and implies that both proteins are able to recruit each other to their respective locations in the absence of other signaling components. The reverse mCherry-MyD88/TIRAP-GFP combination yielded identical results (Figure 7b, first line). In this setting, we tested the respective BB loop point mutants of TIRAP and MyD88. Neither mCherry-MyD88^P200H^ was able to co-localize with WT TIRAP-GFP, nor TIRAP^P125H^-GFP did so with mCherry-MyD88 (Figure 7b), indicating that the integrity of their respective TIR domains was essential for interaction. On the other hand, we visualized together EGFP-TRIF and TRAM-mCherry (not shown), or TRIF-mCherry and TRAM-EGFP (Figure 7c), but no obvious co-localization was detected: TRAM filaments were not decorated with TRIF, and TRIF cytoplasmic spots did not co-stain with TRAM. Occasionally TRIF-mCherry spots were found near TRAM-GFP filaments, but they never overlapped (Figure 7c, hollow arrowheads). However, cells with TRIF-mCherry signal formed shortened TRAM-EGFP filaments or spots (Figure 7c, small arrows), and the characteristic long TRAM-EGFP filaments were only observed in cells lacking red signal (Figure 7c, white arrowheads). This suggests that the presence of TRIF somehow restricts TRAM filament elongation. In any case, the lack of clear TRAM-TRIF co-localization reflects that additional components or cellular processes, that may be necessary for their co-occurrence in cellular compartments, are missing in the yeast model, as compared to the mammalian cell scenario.

### 3.8. TIR-TIR Interactions Involved in SMOC Assembly Can Be Reproduced in Yeast

**Figure 8:**
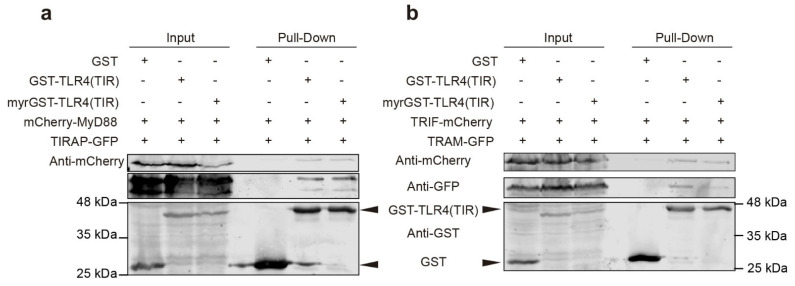
The TIR domain of TLR4 interacts with human TIR adaptors in yeast. YPH499 yeast cells were co-transformed with either GST, GST-TLR4(TIR), or myr-GST-TLR4(TIR) from pEG(KG) derivative plasmids and (**a**) mCherry-MyD88 plus TIRAP-GFP from pYES3 or YCpLG respectively or (**b**) TRIF-mCherry plus TRAM-GFP from pYES3 or YCpLG respectively. The (+) or (-) signs indicate presence or absence, respectively, of the indicated heterologous proteins in the yeast lysates loaded in each electrophoretic lane. Extracts were treated with glutathione agarose beads (GE Healthcare), followed by Western blotting analysis. Input lanes show the whole extract, whereas pull-down lanes display the precipitated proteins. Blots were developed using antibodies anti-mCherry (upper panels), anti-GFP (central panels), and anti-GST (lower panels).

The above results using fluorescent protein fusions of TLR signaling components indicate that protein-protein interactions leading to myddosome and triffosome assembly can be synthetically reproduced in the yeast cell. Such protein-protein interactions were subsequently confirmed biochemically. We performed GST pull-down experiments using as a bait GST-TLR4(TIR), both myristoylated or non-myristoylated to fish either the TIRAP-MyD88 pair or the TRAM-TRIF pair of adaptors. As shown in Figure 8, both GST-TLR4(TIR) and myr-GST-TLR4(TIR), but not GST alone, were able to simultaneously pull-down MyD88 and TIRAP (Figure 8a), in consistence with our microscopy results in which TLR4-TIRAP PM-associated clusters recruit MyD88 (Figure S9a). Peculiarly, TRAM and TRIF were also pulled-down by both GST-TLR4(TIR) fusion versions (Figure 8b), recapitulating results in higher cells as well, despite the lack of co-localization of TRAM and TRIF in our microscopy experiments both in the absence of TLR4(TIR) (see above Figure 7c) or in its presence (Figure S9b). Thus, unlike in the case of MyD88 and TIRAP, TRIF-TRAM interactions may account for the curtail of TRAM filaments in the presence of TRIF, but they are not robust enough to result in co-localization in the absence of additional SMOC elements.

## 4. Discussion

Here, we report expression of components of the human TLR4-dependent myddosome and triffosome SMOCs in the *S. cerevisiae* unicellular eukaryotic model, as well as preliminary studies of their behavior in this heterologous system. With this knowledge we hope to open avenues for synthetic biology platforms that will contribute to explore the function of these complexes at the molecular level.

We previously reported that particular TIR domains of bacterial origin are extremely toxic for yeast due to depletion of NAD^+^ and ATP [42], in consistence with their NAD^+^ hydrolase catalytic activity [57]. In contrast, here we show that human TIR-domain-containing TLR signaling adaptors are tolerated by the yeast cell even at high level, such as those achieved here using the strong inducible *GAL1* promoter. This corroborates the lack of catalytic activity in this set of TIR domains, and presents the yeast model as a feasible platform for studies on their assembly and function. An interesting feature of TIR-domain containing proteins when heterologously produced in yeast is their ability to homopolymerize and assemble long filaments in the cell. We previously reported this phenomenon for *Brucella* BtpA and BtpB TIR domains [42], and show here that the SMOC adaptors TIRAP and, especially, TRAM share this trait. Also, the fact that TIRAP, unlike other proteins with affinity for PtdIns(4,5)P_2_, is excluded from the bud neck area, which is enriched in PtdIns(4,5)P_2_ [58], indicates that TIRAP interferes with the septin mesh there assembled. This observation may imply that TIRAP itself assembles at the PM as a bulky polymeric structure as well, unable to diffuse through the septin barrier. An interesting conclusion that can be derived from our results is that interaction of both TIRAP and TRAM with the PM is necessary for the formation of filaments. This is evidenced by the facts that the TIRAP ×4KA mutant, lacking the positive charges at its N-terminal non-TIR extension that allow interaction with PtdIns(4,5)P_2_ [7] showed up diffuse in the cytosol, instead of producing cytoplasmic filaments, as other TIR domains spontaneously do [42]; and a G2A non-myristoylatable TRAM mutant which cannot attach to membranes also failed to assemble filaments. TRAM^G2A^ diffusion into the cytosol has already been described in mammalian cell lines, where the mutant is no longer able to spread the signal [19].

TRAM develops long filaments along the yeast PM, that indeed rely on TIR-TIR interactions, as we clearly show that a point mutation in a residue at its BB loop, a region essential for TIR-TIR interaction, abrogates filament formation. In consistence with our observations, the TIR domain of TIRAP has been recently shown to assemble into long filaments via self-interaction in vitro, and the TIR domain of TRAM has been suggested to do so as well [10]. Indeed, TRIF and TRAM TIR domains oligomerize and precipitate in solution. Thus, to obtain their crystal structures, they had to be monomerized via the introduction of their corresponding BB loop mutations (TRIF P434H, TRAM C117H), evidencing that an intact BB loop is required for oligomerization [59]. TRAM filaments have not been detected in mammalian cells, and this can be due to different reasons. First, they may not appear at physiological concentrations, and thus yeast overexpression might evidence their intrinsic in vivo self-aggregation ability. Second, TRAM self-assembly may be counteracted by endocytosis. We show here that, in yeast, when filaments cannot form properly, such as in the BB loop mutant C117H TRAM, this protein is found in vacuolar membranes, suggesting that it may be readily endocytosed. Finally, the presence in the cell of TRAM-interacting TIR domains from other proteins, such as those of TLR4(TIR) or TRIF, negatively affects the formation of TRAM filaments, that turn into thick PM-associated patches.

TLR4(TIR)-dependent cluster formation was also observed for the TIRAP adaptor. Furthermore, TLR4-TIRAP PM-associated clusters at the yeast PM were able to recruit MyD88, likely recapitulating in yeast the essential TIR-TIR heterotypic interaction events for myddosome assembly. Of note, TIRAP was also able to recruit MyD88 in the absence of TLR4(TIR), suggesting that TLR4-TIRAP interaction is not a prerequisite for TIRAP-MyD88 interaction. This is in consonance with the observation that, in vitro, the MyD88 TIR domain oligomerizes in a dose-dependent manner in the presence of TIRAP TIR domains, but not in the presence of those of TRAM or TLR4 [10].

An intriguing result is the fact that TRIF and TRAM do not clearly co-localize in the yeast model, despite being co-purified together by TLR4(TIR). This differs from the clear colocalization and interaction of the TIRAP-MyD88 pair in the yeast system, which requires an intact BB loop in any of both elements. A few explanations that account for this result are: (i) a third component is missing in the heterologous system as compared to the mammalian cell, (ii) the N-terminal side of TRIF is preventing it from joining TRAM [60], or (iii) as described for TLR3 signaling [61], TRIF may be only transiently co-localizing with TRAM in the activated mammalian cell, moving quickly to its cytosolic speckle, and thus we are not able to visualize that in *S. cerevisiae*. In any case, we observed that the presence of TRIF limits the ability of TRAM to assemble long filaments, suggesting that some interaction must take place.

Core adaptors MyD88 and TRIF were found in spots inside the yeast cell and did not render any evident filaments, at least within the resolution limits of the fluorescence and confocal microscopes. They were expressed as full-length proteins, with their additional domains having interacting properties too, such as the DD in MyD88 and both the C-terminal RIP homotypic interaction (RHIM) motif and the autoinhibitory N-terminal region in TRIF. The latter has structural similarity to InterFeron-Induced protein with Tetratricopeptide repeats (IFIT) proteins, also involved in protein-protein interactions [62]. These extra motifs may interfere with the ability to assembly into TIR-TIR driven filaments in yeast. We are uncertain about the nature of TRIF cytoplasmic spots in yeast. However, MyD88 spots were clearly associated with ER microdomains, appeared close to mitochondria, and co-localized with the ERMES components Mmm1 and Mdm34. While organelle junctions are gaining interest as signaling hubs, the localization of MyD88 in higher cells remains to be elucidated. Like in our yeast model, MyD88 is known to localize in higher cells in discrete cytoplasmic spots of yet unknown nature when overexpressed. Interestingly, Nishiya et al. [63] reported that localization to those spots did not depend on the TIR domain of MyD88, but on its N-terminal extension, containing the DD and flanking regions, which were essential for both localization and TLR4 signaling. This is consistent with our results in yeast, showing that mutations in the TIR domain did not alter localization. The fact that in the yeast heterologous model MyD88 localizes to membranes matching the ER-mitochondrial junction opens the possibility that membrane contact sites (MCSs) are also important hubs for TLR signaling. Interestingly, orthologs of yeast ERMES components have been found to function at MCSs involving contact of ER membranes not only with mitochondria, but also with endolysosomes for endosome maturation [64]. Moreover, it was recently found that the N-terminal domain of MyD88 efficiently bound phosphatidic acid (PA) in vitro [65]. PA is synthesized in the ER and imported to the mitochondria by the Ups1/Mdm35 lipid transporter to be converted into cardiolipin [66,67]. Thus, it is likely that ER-mitochondria junctions are rich in PA and this accumulation marks a spot for MyD88 localization in the yeast cell.

The hierarchy of myddosome assembly is a matter of controversy. Recent evidence obtained by microcrystal electron diffraction and serial femtosecond crystallography favors a sequential model in which TIRAP provides a platform for the unidirectional assembly of MyD88 oligomers [68], whereas the observation of pre-myddosome scaffolds based on DD interactions have led to the hypothesis that MyD88 is pre-assembled before TLR4 activation [69]. Indeed, ERMES-associated MyD88 clusters observed here in the yeast cell seem to form even when key TIR residues are mutated, so they might be composed of DD-driven ordered structures. However, MyD88 is efficiently recruited to TIRAP PM-associated clusters in the yeast cell, in favor of a sequential assembly model. Overall, the body of results presented here prove that the yeast system can be developed and exploited to help elucidating particular aspects of the assembly and subcellular localization of TLR signaling components. Thus, our work pioneers new and interesting paths for developing humanized yeast models to understand the molecular mechanisms that govern innate immunity and inflammation.

## Supplementary Materials

The following are available online at https://www.mdpi.com/article/10.3390/biom11111737/s1, Figure S1: Expression of human TIR adaptors is tolerated by yeast cells, Figure S2: Colocalization of human TIRAP with distinct membrane compartment markers, Figure S3: Expression of human TIR adaptor mutants in yeast is non-toxic, Figure S4: TRAM-mCherry filaments at the plasma membrane do not coincide with bud scars, Figure S5: Expression of human TLR4 C-terminal region (657-839), containing the TIR domain, is not toxic in yeast, Figure S6: Co-localization of TRIF-mCherry and EGFP-TRIF, Figure S7: Neither MyD88 nor TRIF co-localize with the Golgi marker P4C(SidC), Figure S8: mCherry-MyD88 colocalizes with Hsp-104-mNG labeled stress granules after heat shock at 42 °C, Figure S9: TLR4(TIR)-induced TIRAP clusters recruit MyD88 in yeast, but TLR4(TIR)-induced TRAM clusters do not recruit TRIF, Table S1: *S. cerevisiae* strains used in this work, Table S2: Plasmids generated and used in this work, Table S3: Oligonucleotides used in this work.
