# Supplementary material for: Heterologous Expression and Assembly of Human TLR Signaling Components in Saccharomyces cerevisiae
Source: Biomolecules. 2021 Nov 22;11(11):1737. doi: 10.3390/biom11111737 (PMC8615643; doi:10.3390/biom11111737)
Supplement: Supplementary file 1 [file biomolecules-11-01737-s001.zip › biomolecules-1331951-supplementary.pdf]

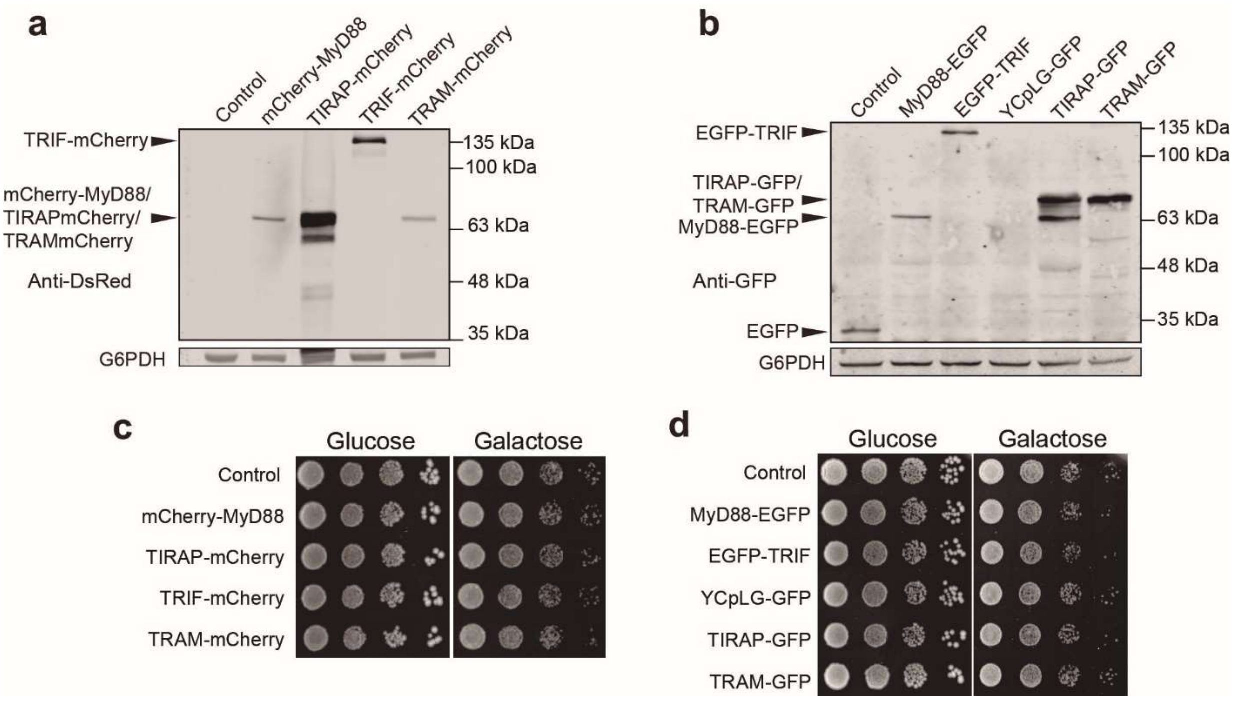

Supplementary Figure S1. Expression of human TIR adaptors is tolerated by yeast cells. (**a, b**) Immunoblots from lysates of cells expressing the indicated mCherry (pYES3) (**a**) or EGFP (pAG425GAL-EGFP)/GFP
(YCpLG) fusions (**b**). Empty plasmids were included as controls. Anti-DsRed (**a**) or anti-GFP (**b**) primary antibodies were used for immunodetection of fusions, and anti-G6PDH as a loading control. Approximate molecular weights are indicated in the figure. The molecular weighs of the constructs are mCherry-MyD88 (60 kDa), TIRAP-mCherry (52 kDa), TRIF-mCherry (103 kDa), TRAM-mCherry (54 kDa),EGFP (29 kDa), EGFP-MyD88 (62 kDa), EGFP-TRIF (105 kDa), GFP (27 kDa), TIRAP-GFP (52 kDa), TRAM-GFP(54 kDa). (**c, d**) Growth under repression (Glucose, left) and induction (Galactose, right) conditions to evaluate effects on yeast growth. None of the expressed proteins caused growth defects in yeast as compared to the empty plasmid controls.

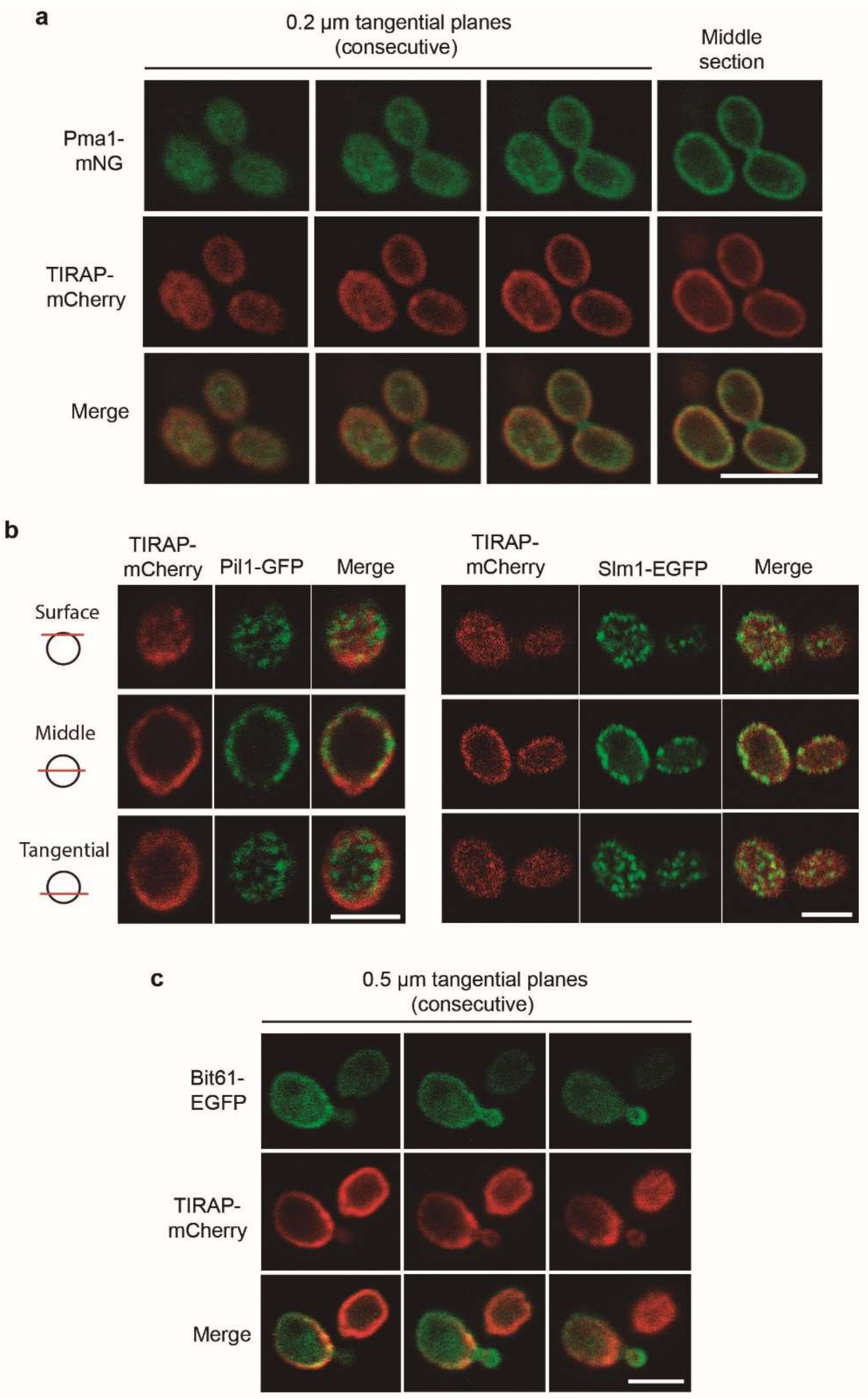

Supplementary Figure S2. Colocalization of human TIRAP with distinct membrane compartment markers. Different confocal planes of a representative cell expressing TIRAP-mCherry from pYES3 and: (**a**) the MCP marker Pma1-mNG (from EVY3 strain), (**b**) the MCC marker Pil1-GFP (from TW110 strain) (left) and the MCC/MCT marker Slm1-EGFP (from pAG413GPD derived plasmid (right), and (**c**) the MCT marker Bit61- EGFP (from pAG-413GPD derived plasmid). Scale bars correspond to 5 µm.

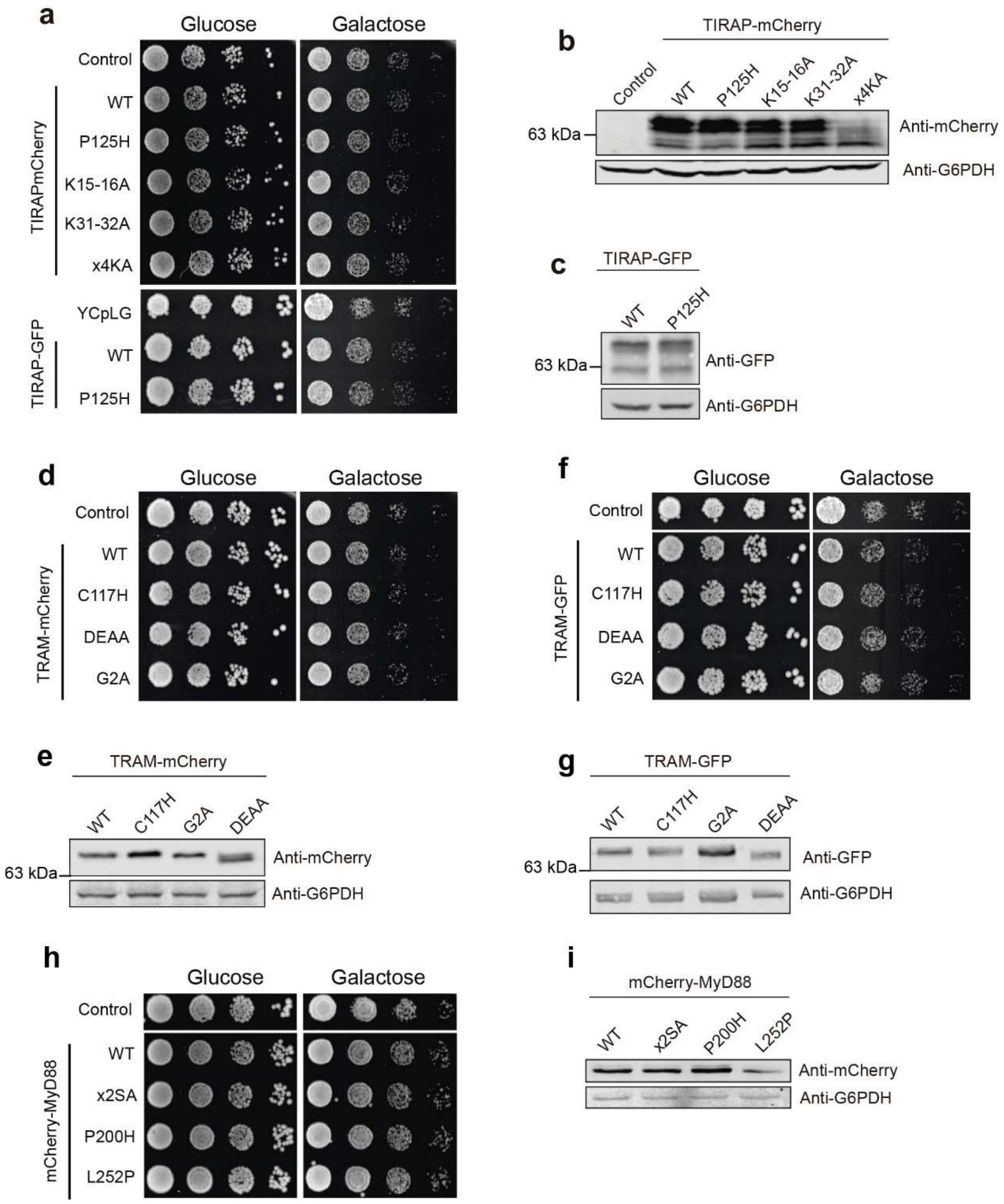

Supplementary Figure S3. Expression of human TIR adaptor mutants in yeast is non-toxic. (**a, d, f, h**) Growth under repression (Glucose, left) and induction (Galactose, right) conditions to evaluate effects on yeast growth. Like the corresponding WT versions, none of the expressed proteins caused growth defects in yeast as compared to the empty vector controls. (**b, c, e, g, i**) Immunoblots from lysates of cells expressing the indicated mCherry or GFP fusions. Anti-DsRed (b, e) or anti-GFP (**c, g**) primary antibodies were used for immunodetection of fusions, and anti-G6PDH as a loading control, as indicated. The molecular weigh of the constructs are indicated on Fig. S1.

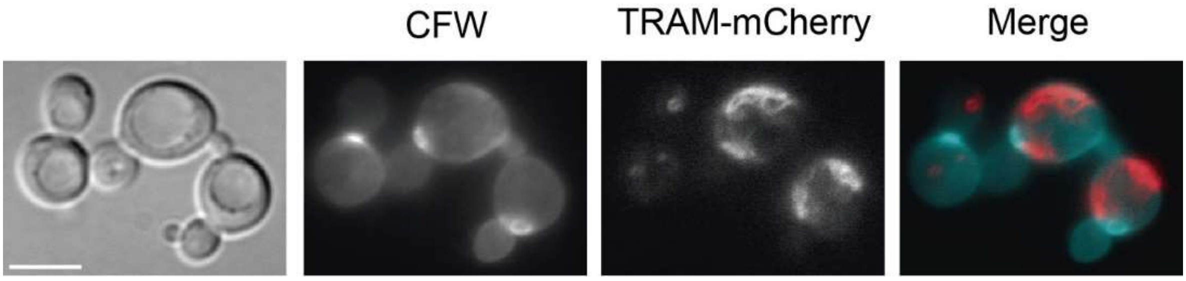

Supplementary Figure S4. TRAM-mCherry filaments in the plasma membrane do not coincide with bud scars. Differential interference and fluorescence microscopy of YPH499 cells transformed with pYES3-TRAM-mCherry and stained with Calcofluor-white. Scale bar represents 5 μm.

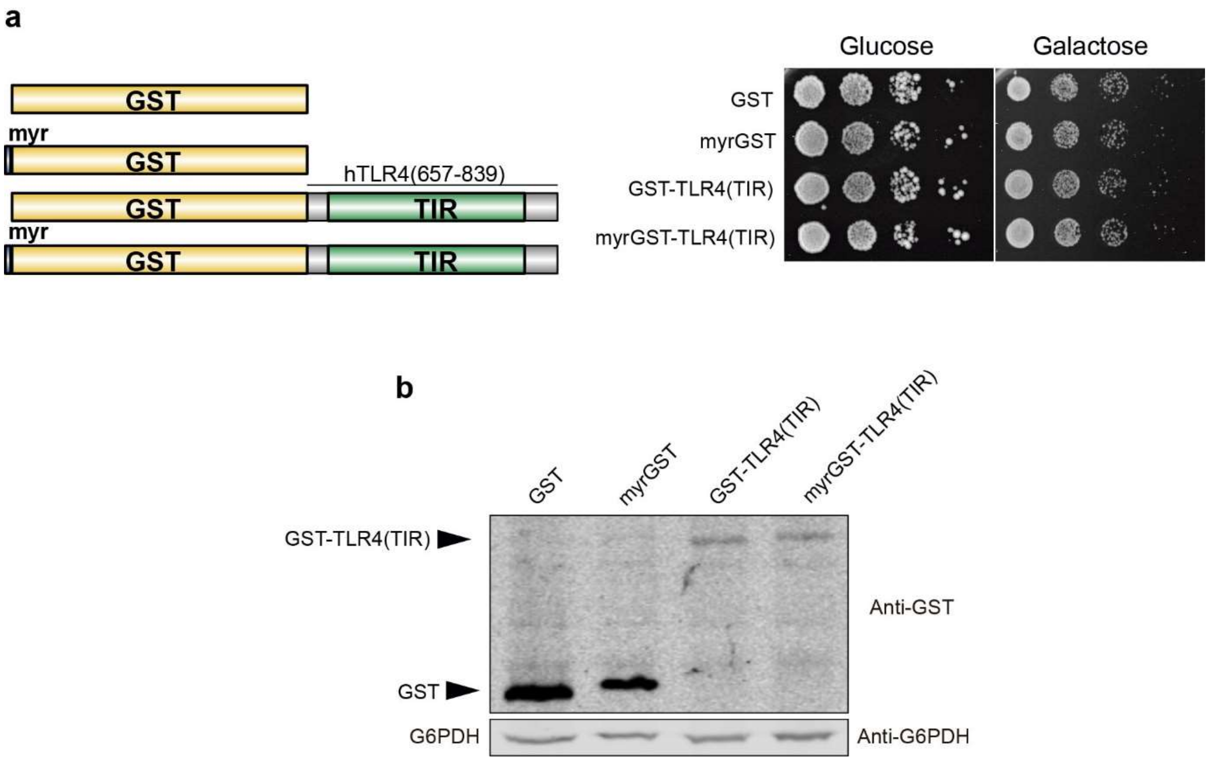

Supplementary Figure S5. Expression of human TLR4 C-terminal region (657-839), containing the TIR domain, is not toxic in yeast. (**a**) Growth under repression (Glucose, left) and induction (Galactose, right) conditions to evaluate effects on yeast growth as compared to the empty vector controls. A scheme of each expressed protein is shown at the left. (**b**) Immunoblot from lysates of cells expressing the indicated GST fusions. An anti-GST primary antibody was used for immunodetection of fusions, and anti-G6PDH as a loading control, as indicated.

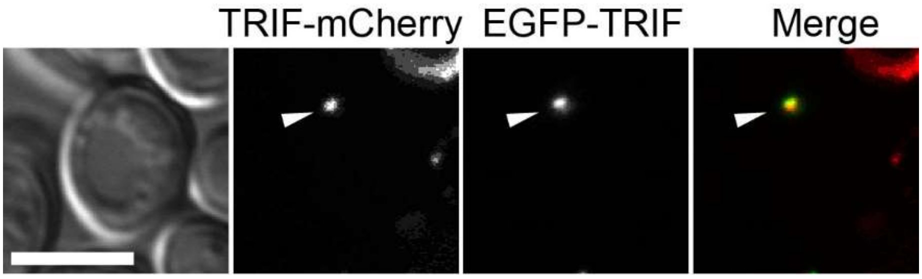

Supplementary Figure S6. Co-localization of TRIF-mCherry and EGFP-TRIF. Differential interference contrast and fluorescence microscopy of YPH499 yeast cells expressing TRIF-mCherry and EGFP-TRIF from plasmids pYES3-TRIF-mCherry and pAG425GAL-EGFP-TRIF respectively. The white arrow indicates a co-localization event. Scale bar represents 5 μm.

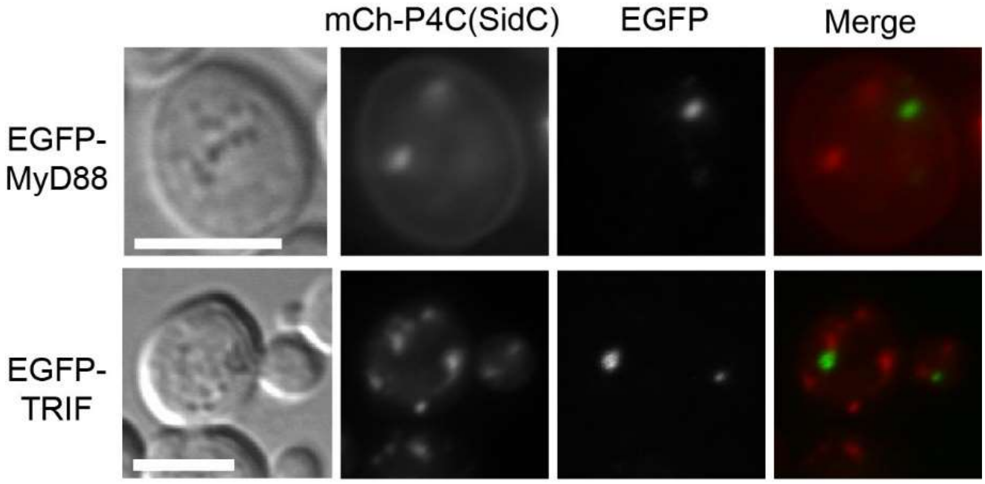

Supplementary Figure S7. Neither MyD88 nor TRIF co-localize with the Golgi. marker P4C(SidC). Fluorescence microscopy of YPH499 yeast cells co-expressing the Golgi marker based on the PtdIns4P marker SidC from *Legionella pneumophila* (plasmid mCherry-P4C(SidC) pESC-TRP) and EGFP-MyD88 from pYES2 (upper panel) or EGFP-TRIF from pAG425GAL (lower panel). Scale bars represent 5 μm.

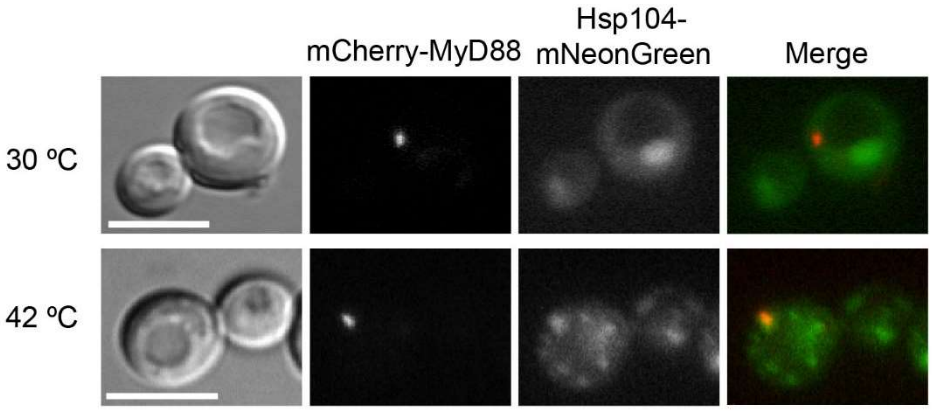

Supplementary Figure S8. mCherry-MyD88 colocalizes with Hsp-104-mNG labeled stress granules after a 42 °C heat shock. Differential interference contrast and fluorescence microscopy of pYES3-mCherry-*MyD88*-transformed LEVY strain heat-shocked or not for 30 min at 42 °C after 5 h of growth under optimal conditions. Scale bar represents 5 µm.

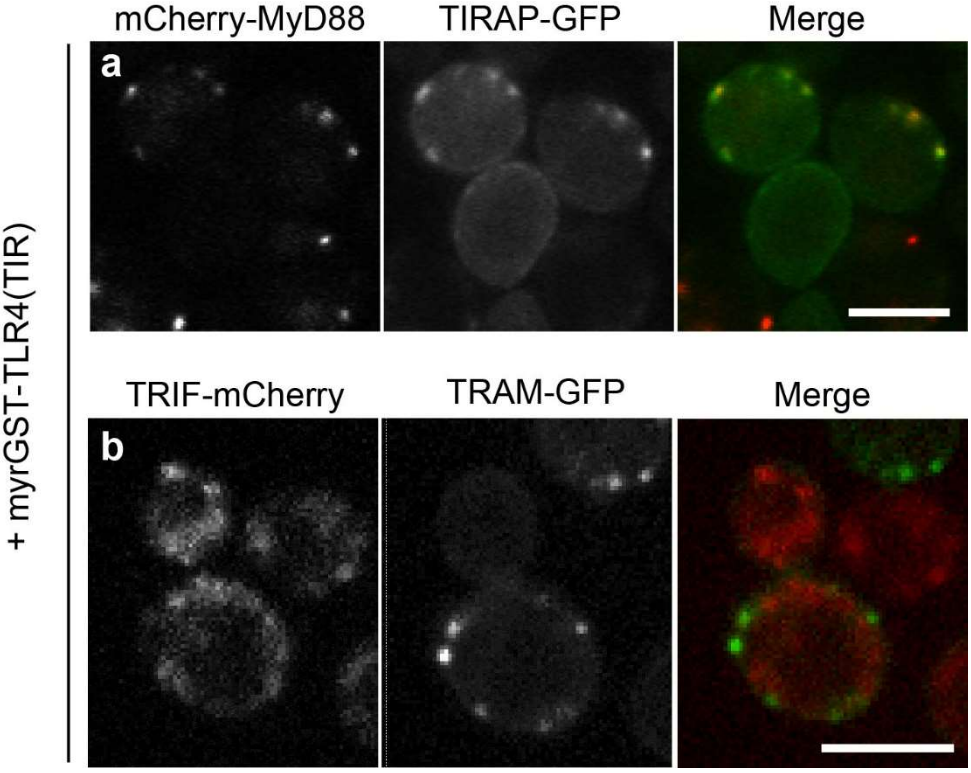

Supplementary Figure S9. TLR4(TIR)-induced TIRAP clusters recruit MyD88 in yeast, but TLR4(TIR)-induced TRAM clusters do not recruit TRIF. Fluorescence (**a**) or laser confocal (**b**) microscopy of YPH499 yeast cells expressing myr-GST-TLR4(TIR), TIRAP-GFP, and mCherry-MyD88 (**a**) or myr-GST-TLR4(TIR), TRAM-EGFP, and TRIF-mCherry (**b**). Scale bars represent 5 µm.

Supplementary Table S1. *S. cerevisiae* strains used in this work.

Strain name	Genotype	From
YPH499	*MATa ura3-52 lys2-801_amber ade2-101_ochre trp1-Δ63 his3-Δ200 leu2-Δ1*	Sikroski et al., 1989[1]
BY4741*trp1*Δ	*MATa his3Δ1 leu2Δ0 met15Δ0 ura3Δ0 trp1Δ::NatMX6*	A gift from A. Sellers (Complutense University, Madrid, Spain)
VHY87	*MATα leu2-3, 112 ura3-52 his4 can1^r TRP1::DsRed-HDEL*	A gift of M. Cyert (Stanford University, CA, USA)[2]
TWY110	*MATa leu2-3,112 trp1-1, can1-100, ura3-1, ade2-1, his3-11,15, PIL1-GFP::HIS3*	A gift from T. C. Walther (Harvard Chan School of Public Health, Boston, MA, USA)[3]
EVY3	*Pma1-mNeonGreen::HygR* Isogenic to BY4741*trp1*Δ	This work
EVY4	*Mdm34-mNeonGreen::HygR* Isogenic to YPH499	This work
EVY5	*Mmm1-mNeonGreen::HygR* Isogenic to YPH499	This work
LEVY1	*Hsp104-mNeonGreen::HygR* Isogenic to YPH499	This work

1. Sikorski, R.S. & Hieter, P. A system of shuttle vectors and yeast host strains designed for efficient manipulation of DNA in Saccharomyces cerevisiae. *Genetics* 122, 19-27 (1989).
2. Heath, V.L., Shaw, S.L., Roy, S. & Cyert, M.S. Hph1p and Hph2p, novel components of calcineurin-mediated stress responses in Saccharomyces cerevisiae. *Eukaryot Cell* 3, 695-704 (2004).
3. Walther, T.C. *et al.* Eisosomes mark static sites of endocytosis. *Nature* 439, 998-1003 (2006).

Supplementary Table S2. Plasmids generated and used in this work.

Plasmid	Description (marker, promoter, type)	Source/Reference
**Cloning vectors and control plasmids**		
pYES2	Control yeast empty vector (*URA3*, *GAL1*, episomal)	Invitrogen
pYES2-GFP	Yeast expression episomal plasmid for N-terminal GFP fusions (*URA3*, *GAL1*, episomal)	[1]
pYES2-mCherry	Yeast expression episomal plasmid for N-terminal mCherry fusions (*URA3*, *GAL1*, episomal)	This work
pYES3	Control yeast empty vector (*TRP1*, *GAL1*, episomal)	Invitrogen
pESC-TRP	Yeast expression empty vector (*TRP1*, *GAL1*, episomal)	Agilent technologies
pYES3-GFP	Yeast expression episomal plasmid for N-terminal GFP fusions (*TRP1*, *GAL1*, episomal)	[2]
pYES3-mCherryCt	Yeast expression episomal plasmid for C-terminal mCherry fusions (*TRP1*, *GAL1*, episomal)	A gift of I. Rodríguez-Escudero (Complutense University, Madrid, Spain)
pYES3-mCherryNt	Yeast expression episomal plasmid for N-terminal mCherry fusions (*TRP1*, *GAL1*, episomal)	[3]
pAG425GAL-EGFP-ccdB	Gateway destination vector (*LEU2*, *GAL1*, episomal)	Addgene Kit # 1000000011 [4]
pAG425GAL-EGFP	Control plasmid from pAG425GAL-EGFP-ccdB bearing the polylinker region from pEG(KG) cloned via Gateway (*LEU2*, *GAL1*, episomal)	This work
YCpLG	Control yeast empty vector (*LEU3*, *GAL1*, centromeric)	A gift from Dr. J. W. Thorner [5]
YCpLG-GFP	Yeast expression centromeric plasmid for C-terminal GFP fusions (*LEU2*, *GAL1*, centromeric)	[6]
pEG(KG)	Yeast expression episomal plasmid for N-terminal GST fusions (*URA3-leu2d*, *GAL1*, episomal)	[7]
myr-pEG(KG)	Modified pEG(KG) for myristoylated GST N-terminal fusions: A myristoilation signal was inserted before GST using InsMyrKG-Up and Lo primers (*URA3-leu2d*, *GAL1*, episomal)	This work
**Yeast expression plasmids**		

pYES3-mCherry-MyD88	Human MyD88 cDNA mCherry fusion for yeast expression (*TRP1*, *GAL1*, episomal)	This work
pYES2-GFP-MyD88	Human MyD88 cDNA GFP fusion for yeast expression (*URA3*, *GAL1*, episomal)	This work
pAG425GAL-MyD88-EGFP	Human MyD88 cDNA EGFP fusion for yeast expression, via gateway cloning (*LEU2*, *GAL1*, episomal)	This work
YCpLG-TIRAP-GFP	Human TIRAP (isoform b) cDNA GFP fusion for yeast expression (*LEU2*, *GAL1*, centromeric)	This work
pYES3-TIRAP-mCherry	Human TIRAP (isoform b) cDNA, mCherry Ct fusion for yeast expression (*TRP1*, *GAL1*, episomal)	This work
pYES2-mCherry-TIRAP	Human TIRAP (isoform b) cDNA, mCherry Nt fusion for yeast expression (*URA3*, *GAL1*, episomal)	This work
pAG425GAL-EGFP-TRIF	Human TRIF cDNA EGFP fusion for yeast expression, via gateway cloning (*LEU2*, *GAL1*, episomal)	This work
pYES3-TRIF-mCherry	Human TRIF cDNA mCherry fusion for yeast expression (*TRP1*, *GAL1*, episomal)	This work
YCpLG-TRAM-GFP	Human TRAM cDNA GFP fusion for yeast expression (*LEU2*, *GAL1*, centromeric)	This work
pYES3-TRAM-mCherry	Human TRAM cDNA mCherry fusion for yeast expression (*TRP1*, *GAL1*, episomal)	This work
pEG(KG)-GST-TLR4-TIR	Human TLR4 C-terminal region cDNA, GST fused for yeast expression (*URA3-leu2d*, *GAL1*, episomal)	This work
pEG(KG)-myrGST-TLR4-TIR	Human TLR4 C-terminal region cDNA, myr-GST fused for yeast expression (*URA3-leu2d*, *GAL1*, episomal)	This work
pLA10H	*S.cerevisiae* Cdc10 fusion to GFP, which was used to visualize the yeast septin ring. (*HIS3*, *MET25*, centromeric)	[8]
pRS426-GFP2XPH(PLCδ)	A fluorescent reporter for PtdIns4,5P_2 (*URA3*, *GAL1*, episomal)	[9]
mCherry-P4C(SidC) pESC-TRP	A mCherry fusion of the PtdIns4P binding region (P4C) from the *Legionella pneumophila* effector SidC (609-775)	This work
YEPlac112-Ilv6-mCherry	A mCherry fusion of the mitochondrial marker Ilv6	[10]

pAP67	Plasmid bearing the mNeonGreen sequence and the Hygromycin Resistance Gene Cassette used for the yeast strains with mNeonGreen-fused proteins.	A gift from A. Pérez and J. W. Thorner
pAG413GPD-Slm1-EGFP	A EGFP fusion of the MCT component Slm1(*HIS3*, *GPD*, centromeric)	This work
pAG413GPD-Bit61-EGFP	A EGFP fusion of the MCT component Bit61 (*HIS3*, *GPD*, centromeric)	This work
BG1805-Slm1	Plasmids bearing the *attB* sequences, that were used for subcloning into Gateway destination vectors	Yeast ORF Collection (Dharmacon)
BG1805-Bit61		
**Site-directed mutants**		
pYES3-mCherry-MyD88 x2SA	MyD88 mutant on two phosphorylatable serines, S242A S244A (TRP1, GAL1, episomal)	This work
pYES3-mCherry-MyD88 P200H	MyD88 mutant on the BB loop P200H (TRP1, GAL1, episomal)	This work
pYES3-mCherry-MyD88 L252P	MyD88 gain-of-function, oncogenic mutant (TRP1, GAL1, episomal)	This work
YCpLG-TIRAP P125H-GFP	TIRAP mutant on the BB loop P125H (LEU2, GAL1, centromeric)	This work
pYES3-TIRAP P125H mCherry	TIRAP mutant on the BB loop proline (TRP1, GAL1, episomal)	This work
pYES3-TIRAP K15-16A mCherry	TIRAP mutant on two PM targeting lysines K15A K16A (TRP1, GAL1, episomal)	This work
pYES3-TIRAP K31-32A mCherry	TIRAP mutant on two other PM targeting lysines K31A K32A (TRP1, GAL1, episomal)	This work
pYES3-TIRAP x4KA mCherry	TIRAP mutant on the4 PM targeting lysines K15A K16A K31A K32A (TRP1, GAL1, episomal)	This work
pYES3-TRAM C117H-mCherry	TRAM mutant on the BB loop C117H (TRP1, GAL1, episomal)	This work
pYES3-TRAM G2A-mCherry	TRAM lacking myristoylation signal G2A (TRP1, GAL1, episomal)	This work
pYES3-TRAM DEAA-mCherry	TRAM without an acidic motif D91A E92A (TRP1, GAL1, episomal)	This work
YCpLG-TRAM C117H-GFP	TRAM mutant on the BB loop C117H (LEU2, GAL1, centromeric)	This work

YCpLG-TRAM G2A-GFP	TRAM lacking myristoylation signal G2A (LEU2, GAL1, centromeric)	This work
YCpLG-TRAM DEAA-GFP	TRAM without an acidic motif D91A E92A (LEU2, GAL1, centromeric)	This work
pAG425GAL-EGFP-TRIF P434H	TRIF mutant on the BB loop P434H (LEU2, GAL1, episomal)	This work

1. Rodríguez-Escudero, I., Andrés-Pons, A., Pulido, R., Molina, M. & Cid, V.J. Phosphatidylinositol 3-kinase-dependent activation of mammalian protein kinase B/Akt in Saccharomyces cerevisiae, an in vivo model for the functional study of Akt mutations. *J Biol Chem* **284**, 13373-13383 (2009).
2. Coronas-Serna, J.M. *et al.* The TIR-domain containing effectors BtpA and BtpB from Brucella abortus impact NAD metabolism. *PLoS Pathog* **16**, e1007979 (2020).
3. Rodríguez-Escudero, I., Fernández-Acero, T., Cid, V.J. & Molina, M. Heterologous mammalian Akt disrupts plasma membrane homeostasis by taking over TORC2 signaling in Saccharomyces cerevisiae. *Sci Rep* **8**, 7732 (2018).
4. Alberti, S., Gitler, A.D. & Lindquist, S. A suite of Gateway cloning vectors for high-throughput genetic analysis in Saccharomyces cerevisiae. *Yeast* **24**, 913-919 (2007).
5. Bardwell, L., Cook, J.G., Zhu-Shimoni, J.X., Voora, D. & Thorner, J. Differential regulation of transcription: Repression by unactivated mitogen-activated protein kinase Kss1 requires the Dig1 and Dig2 proteins. *Proceedings of the National Academy of Sciences* **95**, 15400-15405 (1998).
6. Rodríguez-Escudero, I., Rotger, R., Cid, V.J. & Molina, M. Inhibition of Cdc42-dependent signalling in Saccharomyces cerevisiae by phosphatase-dead SigD/SopB from Salmonella typhimurium. *Microbiology* **152**, 3437-3452 (2006).
7. Mitchell, D.A., Marshall, T.K. & Deschenes, R.J. Vectors for the inducible overexpression of glutathione S-transferase fusion proteins in yeast. *Yeast* **9**, 715-722 (1993).
8. Cid, V.c.J., Adamiková, L., Sánchez, M., Molina, M.a. & Nombela, C. Cell cycle control of septin ring dynamics in the budding yeast. *Microbiology* **147**, 1437-1450 (2001).
9. Stefan, C.J., Audhya, A. & Emr, S.D. The yeast synaptojanin-like proteins control the cellular distribution of phosphatidylinositol (4,5)-bisphosphate. *Mol Biol Cell* **13**, 542-557 (2002).
10. Fernández-Acero, T. *et al.* Expression of Human PTEN-L in a Yeast Heterologous Model Unveils Specific N-Terminal Motifs Controlling PTEN-L Subcellular Localization and Function. *Cells* **8** (2019).

Supplementary Table S3. Oligonucleotides used in this work.

Primer	Sequence (5′->3′)	Purpose
**Cloning primers**		
MyD88-Up	5′-ttggatccatggctgcaggaggtcccgg-3′	To amplify hMyD88 and clone it into pYES3-mCherryNt and pYES2-GFP (*Bam*HI-*Eco*RI)
MyD88-Lo	5′-ttgaattctcagggcagggacaaggccttgg-3′	
attB1-MyD88Ct2	5′-ggggacaagtttgtacaaaaaagcaggcttcaccatggctgcaggaggtcc-3′	To amplify hMyD88 to clone it C-terminally tagged via gateway cloning in pAG425GAL1-ccdB-EGFP
attB2-MyD88Ct	5′-ggggaccactttgtacaagaaagctgggtggggcagggacaaggcc-3′	
TIRAP-*Bam*HI-Up	5′-ttggatccatggcatcatcgacctccct-3′	To amplify hTIRAP (isoform b) and clone it into YCpLG-GFP (*Bam*HI-*Bam*HI) and pYES3-mCherryCt (*Hind*III-*Bam*HI) (Ct fusions) and pYES2-mCherry (*Bam*HI-*Bam*HI) (Nt fusions)
TIRAP-*Hind*III-Up	5′-ttaagcttatggcatcatcgacctccct-3′	
TIRAP- LoCt	5′-ttggatccaagtagatcagatactgtagc-3′	
TIRAP- UpNt	5′-ggggatccatggcatcatcgacctccc-3′	
TIRAP-LoNt	5′-ggggatcctcaaagtagatcagatactgtag-3′	
TRIF-*Hind*III-Up	5′-ttaagcttatggcctgcacaggcccatc-3′	To amplify hTRIF and clone it into pYES3-mCherryCt (*Hind*III-*Hind*III)
TRIF-*Hind*III-Lo	5′-ttaagcttttctgcctcctgcgtcttgtc-3′	
TRIF-Nt-attB1-Up	5′-ggggacaagtttgtacaaaaaagcaggcttcatggcctgcacaggcccatca-3′	To amplify hTRIF to clone it N-terminally tagged via gateway cloning in pAG425GAL1-EGFP-ccdB
TRIF-Nt-attB2-Lo	5′-ggggaccactttgtacaagaaagctgggttttatcattctgcctcctgcgtc-3′	
TRAM-*Bam*HI-Up	5′-ttggatccatgggtatcgggaagtcta-3′	To amplify hTRAM and clone it into YCpLG-GFP and pYES3-mCherryCt (*Bam*HI-*Bam*HI)
TRAM-*Bam*HI-Lo	5′-ttggatccggcaataaattgtctttgtac-3′	
TLR4-TIR-Up	5′-ttggatcccacctgatgcttcttgctg-3′	To amplify hTLR4 (657-839) and clone it into pEG(KG) and myr-pEG(KG) (*Bam*HI-*Hind*III)
TLR4-TIR-Lo	5′-ttaagctttcagatagatgttgcttcctg-3′	
attB1-polyKG	5′-ggggacaagtttgtacaaaaaagcaggcttcggatccccgggaatttcc-3′	To amplify the pEG(KG) polylinker sequence used as "empty" vector in Gateway cloning
attB2-polyKG	5′-ggggaccactttgtacaagaaagctgggtttaaagcttgagctcgagtcg-3′	
Fw-PolyKGFCt	5′-ggggacaagtttgtacaaaaaagcaggcttcaccatgggatccccgggaatttcc-3′	To introduce the Kozak sequence and a start codon

Rv-PolyKGFCt	5′-ggggaccactttgtacaagaaagctgggtgaagcttgagctcgagtcg-3′	in the pEG(KG) polylinker sequence in order to express the C terminal tags of the control vectors
Cherry-*Hind*III-Up	5′-ggaagcttatggtgagcaagggcgagga-3′	To generate pYES2-mCherry vector for N-terminal fusions
Cherry-*Bam*HI-Lo	5′-ggggatcccttgtacagctcgtccatgc-3′	
Cherry-*Bam*HI-Up	5′- cgggatccatggtgagcaagggcgag-3′	To clone the mCherry fused to P4C domain of SidC (609-776) (*Bam*HI-*Nhe*I), into pESC-TRP vector
SidC(609-775)-Lo	5′- ctagctagcttagaattcaattgcttcatcattg-3′	
FusCherry-SidC(609-775)-Up	5′- gacgagctgtacaagggagctggtgcatgcaaatattcctccaagccattat-3′	To fuse mCherry to the P4C domain of SidC via overlapping PCR.
FusCherry-SidC(609-775)-Lo	5′- cttggaggaatatttgcatgcaccagctcccttgtacagctcgtccatgc-3′	
Pma1mNG Fw	5′-aaagagtctctactcaacacgaaaaggaaacctctggtagttctggtggtatgg-3′	To amplify mNeonGreen cassette and insert it downstream the respective ORFs in their genomic loci for a C-terminal fusion.
Pma1mNG Rv	5′-tgtgacaaaaattatgattaaatgctacttcaacaggaactggatggcggcgttag-3′	
Mmm1mNG Fw	5′-accaagtatgtggccacgtagtaaaaatacgagagaagaaaagcctacagagttatctggtagttctggtggtatgg-3′	
Mmm1mNG Rv	5′-aaatgaggcagagaagataggaaaaagatagaacaaaaaatttgtacataaatatactggatggcggcgttag-3′	
Mdm34mNG Fw	5′-agaaccttcaaataactggaaatggggcatggaggatagccccccaccatatcattctggtagttctggtggtatgg-3′	
Mdm34mNG Rv	5′-tttacatcggagagtatgtatttgtgtagttatgtacttagatatgtaacttaatactggatggcggcgttag- 3′	
Hsp104mNG Fw	5′-Cacgttaggtgatgacgataatgaggacagtatggaaattgatgatgacctagattctggtagttctggtggtatgg- 3′	
Hsp104mNG Rv	5′-tatattatattactgattcttgttcgaaagtttttaaaaatcacactatattaaaactggatggcggcgttag-3′	

mNGComprob Rv	5′- ccataccaccagaactacc- 3′	To check by colony PCR the correct C-terminal fusion of the mNeonGreen tag in the genomic loci.
Mmm1-C-Fw	5′- gagaatgaatccaccgaaacg- 3′	
Mdm34-C-Fw	5′- attatcacgcagcccaagtc- 3′	
Hsp104-C-Fw	5′- ggtcatgggtgctgttagg- 3′	
**Mutagenic primers**		
InsMyrKG-Up	5′-ctcatgggaactagtaagtcttcccctatactaggttattggaaaatt-3′	Insertion of the myristoylation signal before GST in pEG(KG)
InsMyrKG-Lo	5′-ggaagacttactagttcccatgagctcgaattgatccggtaat-3′	
MyD88 x2SA-Up	5′-gcactcgccctcgctccaggtgcccatcagaagc-3′	To generate MyD88 S242A S244A mutant
MyD88 x2SA-Lo	5′-ctggagcgagggcgagtgcaaatttggtctggaagtcaca-3′	
MyD88 PH-Up	5′-gatgtcctgcatggcacctgtgtctggtcta-3′	To generate MyD88 P200H BB loop mutant
MyD88 PH-Lo	5′-ggtgccatgcaggacatcgcggtcag-3′	
MyD88 LP-Up	5′-cagaagcgaccgatccccatcaagtacaag-3′	To generate MyD88 L252P oncogenic mutant
MyD88 LP-Lo	5′-ggggatcggtcgcttctgatgggca-3′	
TIRAP PH-Up	5′- caacccacggcggcgctatagtgtccg-3′	To generate TIRAP P125H BB loop mutant
TIRAP PH-Lo	5′- gccgccgtgggttgcatcccggagtt-3′	
TIRAP K15-16A-Up	5′- cggcctgcggcgcctctaggcaagatgg-3′	To generate TIRAP K15A K16A mutant
TIRAP K15-16A-Lo	5′- tagaggcgccgcaggccgagagccagga-3′	
TIRAP K31-32A-Up	5′- ctgctggcggcgcccaagaagaggccc-3′	To generate TIRAP K31A K32A mutant
TIRAP K31-32A-Lo	5′- ttgggcgccgccagcagggtctgcctg-3′	
TRAM C117H-Up	5′- tgagatgccacatggcagacagcatttacag-3′	To generate TRAM C117H BB loop mutant
TRAM C117H-Lo	5′- gctgtctgccatgtggcatctcagcaaagat-3′	
TRAM G2A-Up	5′-ggatccatggctatcgggaagtctaaaataaattcct-3′	To generate TRAM G2A mutant in YCplG and pYES3 vectors
TRAM G2A pYES3-Lo	5′- cttcccgatagccatggatccgagctc-3′	

TRAM G2A-YCpLG-Lo	5′- gacttcccgatagccatggatccggttc-3′	
TRAM DEAA-Up	5′-gatgacacagctgcagccctcagagtccag-3′	To generate TRAM D91A E92A mutant in YCplG and pYES3 vectors
TRAM DEAA-Lo	5′-tctgagggctgcagctgtgtcatcttctgcatg-3′	
TRIF P434H-Up	5′- ggtgcacgggcgcggggagctga-3′	To generate TRIF P434H BB loop mutant
TRIF P434H-Lo	5′- cgcccgtgcacctggaaatcctcgca-3′	
